# Real-world treatment and retreatment patterns and outcomes in patients with advanced or metastatic non-small cell lung cancer following nivolumab monotherapy in second line or later in France: an I-O Optimise analysis

**DOI:** 10.3389/fonc.2025.1526931

**Published:** 2025-02-20

**Authors:** Grégoire Justeau, Christos Chouaid, Didier Debieuvre, Clarisse Audigier-Valette, Xavier Quantin, Hervé Léna, Lise Bosquet, Nicolas Girard, Minouk J. Schoemaker, Marta Mella, Bárbara Pinto Correia, Caroline Rault, Melinda J. Daumont, John R. Penrod, Adam Lee, Maurice Pérol

**Affiliations:** ^1^ Department of Pneumology, Angers University Hospital, Angers, France; ^2^ Department of Pneumology and Thoracic Oncology, Centre Hospitalier Intercommunal de Créteil, Créteil, France; ^3^ Department of Pneumology, Groupe Hospitalier de la Region de Mulhouse Sud-Alsace (GHRMSA), Mulhouse, France; ^4^ Department of Thoracic Oncology, Ste Musse Hospital, Toulon, France; ^5^ Montpellier Cancer Institute, Inserm U1194, University of Montpellier, Montpellier, France; ^6^ Department of Pneumology, University Hospital, Rennes, France; ^7^ Health Data and Partnerships Department, Unicancer, Paris, France; ^8^ Department of Pneumology and Thoracic Oncology, Curie Institute, Paris, France; ^9^ University of Versailles Saint-Quentin-en-Yvelines (UVSQ), Paris Saclay University, Versailles, France; ^10^ Global Database Studies, IQVIA, Amsterdam, Netherlands; ^11^ Global Database Studies, IQVIA, Milan, Italy; ^12^ Global Database Studies, IQVIA, Porto Salvo, Portugal; ^13^ Epidemiology, Data-Gnosis, Rennes, France; ^14^ Worldwide HEOR, Bristol Myers Squibb, Brussels, Belgium; ^15^ Worldwide HEOR, Bristol Myers Squibb, Princeton, NJ, United States; ^16^ Worldwide HEOR, Bristol Myers Squibb, Uxbridge, United Kingdom; ^17^ Department of Thoracic Oncology, Léon Bérard Cancer Center, Lyon, France

**Keywords:** real-world, immunotherapy, NSCLC, retreatment, rechallenge, PD-L1 expression

## Abstract

**Introduction:**

This study describes treatment and retreatment patterns and outcomes in patients in France following nivolumab as a second-line or later (2L+) treatment in locally advanced or metastatic non-small cell lung cancer (LAM NSCLC).

**Materials and methods:**

This analysis included adults with tumor, node, metastasis stage IIIB–IV NSCLC (as defined in the 7th or 8th edition American Joint Committee on Cancer/Union for International Cancer Control) treated with nivolumab monotherapy in 2L+ using data from the retrospective Epidemiological-Strategy and Medical Economics Lung Cancer database. The inclusion period was from January 1, 2015, to September 30, 2020, with a follow-up until September 30, 2021. Analyses were stratified according to the duration of index nivolumab treatment and tumor programmed death ligand 1 expression levels.

**Results:**

In total, the study included 4,001 patients (68% male; mean age [standard deviation] at index date, 63.6 [9.7] years) with a median follow-up of 34.3 months. The median nivolumab duration was 2.5 months (interquartile range, 1.4–6.3). The median overall survival (OS) from nivolumab initiation was 10.2 months (95% confidence interval [CI], 9.6–10.8). The median real-world progression-free survival and time to treatment discontinuation or death (95% CI) were 2.2 (2.1–2.3) and 2.7 (2.5–2.8) months, respectively. In total, 2,985 (74.6%) patients discontinued index nivolumab treatment: 226 (7.6% of discontinuers) received a further immune checkpoint inhibitor (ICI; 12.3% of discontinuers receiving further systemic treatment), and 1,604 (53.7%) received chemotherapy and/or targeted therapy. The proportion of ICI-retreated patients was the highest among those with the longest index treatment duration (15.8% among discontinuers receiving ≥26 weeks’ index nivolumab). The median OS from retreatment was longer in the resumption (ICI restart without another therapy for ≥6 weeks) compared with the rechallenge (ICI restart following non-ICI therapy) patient subgroup.

**Conclusion:**

Few patients with LAM NSCLC in France received ICI retreatment following index nivolumab discontinuation, but the proportion increased with a longer duration of index nivolumab.

## 1 Introduction

Lung cancer is a leading global health issue, with Europe reporting more than 484,000 new cases in 2022 and almost 50,000 annually in France, ranking it fourth in national cancer incidence (1). More than one-half of lung cancer diagnoses in patients in France are stage IV at diagnosis, contributing to its status as the leading cause of cancer death in France, responsible for approximately 20% of deaths (1, 2). Globally, non-small cell lung cancer (NSCLC) accounts for 85%–90% of lung cancer cases (3). Treatment has advanced and evolved over the past decade from platinum-based chemotherapy to include targeted therapies and immune checkpoint inhibitors (ICIs), including treatments targeting the programmed cell death protein-1 receptor and its ligand, programmed death ligand 1 (PD-L1), improving outcomes for patients with NSCLC, especially those without oncogenic mutations (4–6).

ICIs were first introduced for treating locally advanced or metastatic (LAM) NSCLC following the demonstration of improved overall survival (OS) compared with docetaxel after disease progression on first-line (1L) therapy, leading to nivolumab’s availability in France since 2015 (7–9). Recent pooled data from the pivotal Phase 3 randomized trials CheckMate 017 (squamous NSCLC) and CheckMate 057 (non-squamous NSCLC) have shown 5-year OS rates of nivolumab as second-line (2L) therapy at 13.4% versus 2.6% for docetaxel (10). In Europe, pembrolizumab was approved as a 2L therapy for patients with PD-L1 ≥ 1% in 2016 and as 1L monotherapy for patients with PD-L1 ≥ 50% in 2017 (11). Subsequent evolution of ICI use in Europe from 2L to 1L, regardless of PD-L1 expression, began with the approval of pembrolizumab with chemotherapy in 2018 (11) followed in 2020 by the combination of nivolumab, ipilimumab, and chemotherapy for patients without specific genetic mutations (8, 12, 13).

Real-world data show increasing use of immunotherapy in the years following its introduction in the 2L and later and then as a 1L treatment for advanced NSCLC in France (9, 14). Survival outcomes from real-world use of nivolumab in 2L align with those reported in randomized controlled trials, with 1-year survival rates of approximately 40%–50% (9, 15, 16). Understanding the characteristics and treatment patterns of patients receiving 2L or later (2L+) nivolumab is crucial, as real-world insights can inform the application of these therapies beyond clinical trial settings.

After disease progression and ICI discontinuation, retreatment with ICIs is an option for advanced NSCLC (17). The real-world French UNIVOC study, reporting on outcomes in patients commencing nivolumab between 2015 and 2016 (prior to ICI availability for 1L use), reported a median 2L+ nivolumab treatment duration of 2.8 months and a median OS of 11.5 months (18). Following discontinuation, 29% of patients received a second programmed cell death protein-1 receptor inhibitor course, with better OS outcomes observed in those who had a longer initial treatment duration.

This work utilizes the I-O Optimise multinational research program, leveraging the Epidemiological-Strategy and Medical Economics Lung Cancer (ESME-LC) data to study 2L+ nivolumab use in LAM NSCLC in France (19–21). The study period includes nivolumab’s initial approval to post-1L ICI approvals and aims to understand shifts in the 2L+ patient population and treatment outcomes. This analysis also aims to enhance knowledge of long-term treatment patterns and patient responses to 2L+ ICIs, in addition to retreatment patterns and associated survival outcomes.

## 2 Materials and methods

### 2.1 Study design

This retrospective cohort study included patients diagnosed with LAM NSCLC and treated with nivolumab as a 2L+ between 2015 and 2020 based on the ESME-LC data source in France (NCT03848052). Launched in 2014, the ESME-LC program is a large-scale academic initiative aimed at centralizing longitudinal real-life data from multiple cancer centers and public hospitals. At the time of the analysis, 30 sites were contributing to the ESME-LC database, including 18 French comprehensive cancer centers and 12 public hospitals. These sites were selected as representative of the French population and the French Healthcare system in the treatment of advanced and metastatic lung cancer (9). The patients included in the ESME-LC database at participating hospitals were all treated for lung cancer with radiotherapy or any systemic cancer therapy or had a diagnosis of metastatic lung cancer. It is estimated that the ESME-LC database accounts for approximately 12%–15% of all patients diagnosed with lung cancer in France between 2015 and 2022. The selection process for site participation allows retrospective compilation of a comprehensive list of patients treated for lung cancer at the participating hospitals.

Patients were followed up from the index date to the earliest of the following events: the end of available data (last contact or last patient status update before the study period concluded), known exit from the data source, or death. The follow-up period started from January 1, 2015, to September 30, 2021, allowing by design a minimum follow-up of 1 year and a maximum follow-up of 6 years for included patients.

Index date was defined as the start of initial nivolumab as a 2L+ therapy (index nivolumab treatment).

Retreatment was defined as the start of subsequent IO after index nivolumab treatment.

This study was conducted in accordance with the International Society for Pharmacoepidemiology Guidelines for Good Pharmacoepidemiology Practices and applicable regulatory requirements. The applicable legal provisions were observed. In compliance with French regulations, the ESME-LC data platform was authorized by the French data protection authority and managed by Unicancer in accordance with current best practice guidelines. Owing to the study design (non-interventional, retrospective, descriptive study using anonymized patient data), the need for informed patient consent was waived, but all patients approved the use of their data.

### 2.2 Study population and study period

This study included all adult patients (age ≥18 years at diagnosis) with histologically confirmed stage IIIB-IV LAM NSCLC (as defined in either the 7th or 8th edition American Joint Committee on Cancer/Union for International Cancer Control, *de novo* or relapsed) (22, 23) treated with nivolumab monotherapy in 2L+ between January 1, 2015, and September 30, 2020, in a center of care participating in the ESME-LC database. Patients with a primary malignancy in the 5 years prior to the date of lung cancer diagnosis were excluded.

### 2.3 Stratifications and subgroups

Analyses were conducted in the overall study population and by various stratification or subgroups according to i) duration of index nivolumab treatment, ii) nivolumab treatment discontinuation status at landmark timepoints (continued/discontinued), iii) PD-L1% expression level, and iv) type of retreatment.

#### 2.3.1 Duration of index nivolumab treatment

Patient demographics, clinical characteristics, and treatment patterns were described according to subgroups of index nivolumab treatment duration, as follows: <13 weeks, 13–25 weeks, 26–38 weeks, 39–51 weeks, 52–103 weeks, and ≥104 weeks. Owing to small patient numbers in subgroup analyses, in the retreatment analysis, initial nivolumab duration was grouped into <13 weeks, 13–25 weeks, and ≥26 weeks.

#### 2.3.2 Nivolumab treatment discontinuation status

Nivolumab treatment discontinuation status (continued or discontinued) was used as a stratification to analyze OS and progression-free survival (PFS) in patients achieving survival to prespecified landmarks from nivolumab initiation.

#### 2.3.3 PD-L1% expression level

For stratification of OS and PFS from nivolumab initiation, PD-L1% expression was <1% or “negative”, ≥1% expression or “positive”, or unknown, with PD-L1 test results based on tumor-activated cell score (%).

#### 2.3.4 Retreatment type

Among patients who discontinued index nivolumab and were retreated, the retreatment groups were defined as follows according to the type of treatment they received:

1. Chemotherapy and/or tyrosine kinase inhibitor (TKI)—being retreated with systemic anti-cancer therapy (SACT) agent outside of ICI therapies (i.e., chemotherapy and/or TKI),
2. ICI rechallenge—being retreated with a non-ICI SACT and a subsequent ICI,
3. ICI resumption—being retreated directly with an ICI after a treatment break of >6 weeks (i.e., equivalent to three missed treatment cycles; with no other SACT during the treatment interval), and
4. ICI switch—being retreated with ICI (other than nivolumab) after a treatment break of ≤6 weeks.

Analysis of OS and patient characteristics was conducted using a retreatment-type subgroup.

### 2.4 Statistical analysis

Patient demographics, clinical characteristics, and treatment patterns were analyzed using descriptive statistics.

Continuous variables were represented by the mean, standard deviation, median, and first and third quartiles. Categorical variables were represented by the number and percentage of patients in each category. For all appropriate statistics, 95% confidence intervals (CIs) were presented.

Real-world OS, PFS, and time to treatment discontinuation or death (TTDD) were estimated using a Kaplan–Meier methodology, with a number of patients still at risk at specific timepoints from nivolumab treatment initiation. Analyses were conducted overall and by PD-L1% expression level.

Landmark survival was also estimated from the time of reaching specific survival landmarks (13, 26, 39, and 52 weeks from nivolumab treatment initiation) using a Kaplan–Meier approach. This analysis was performed by nivolumab discontinuation status at the time of the respective landmark.

OS in the retreatment subgroup (in patients who resumed or were rechallenged) was analyzed using the Kaplan–Meier methodology and assessed from the date of retreatment with ICI therapy according to the method of retreatment stratification.

A sensitivity analysis was conducted by assessing patients who received index nivolumab in the 2L only, separate from patients who received it as a third-line or later (3L+) therapy.

## 3 Results

### 3.1 Patient demographics and characteristics

**Figure 1:**
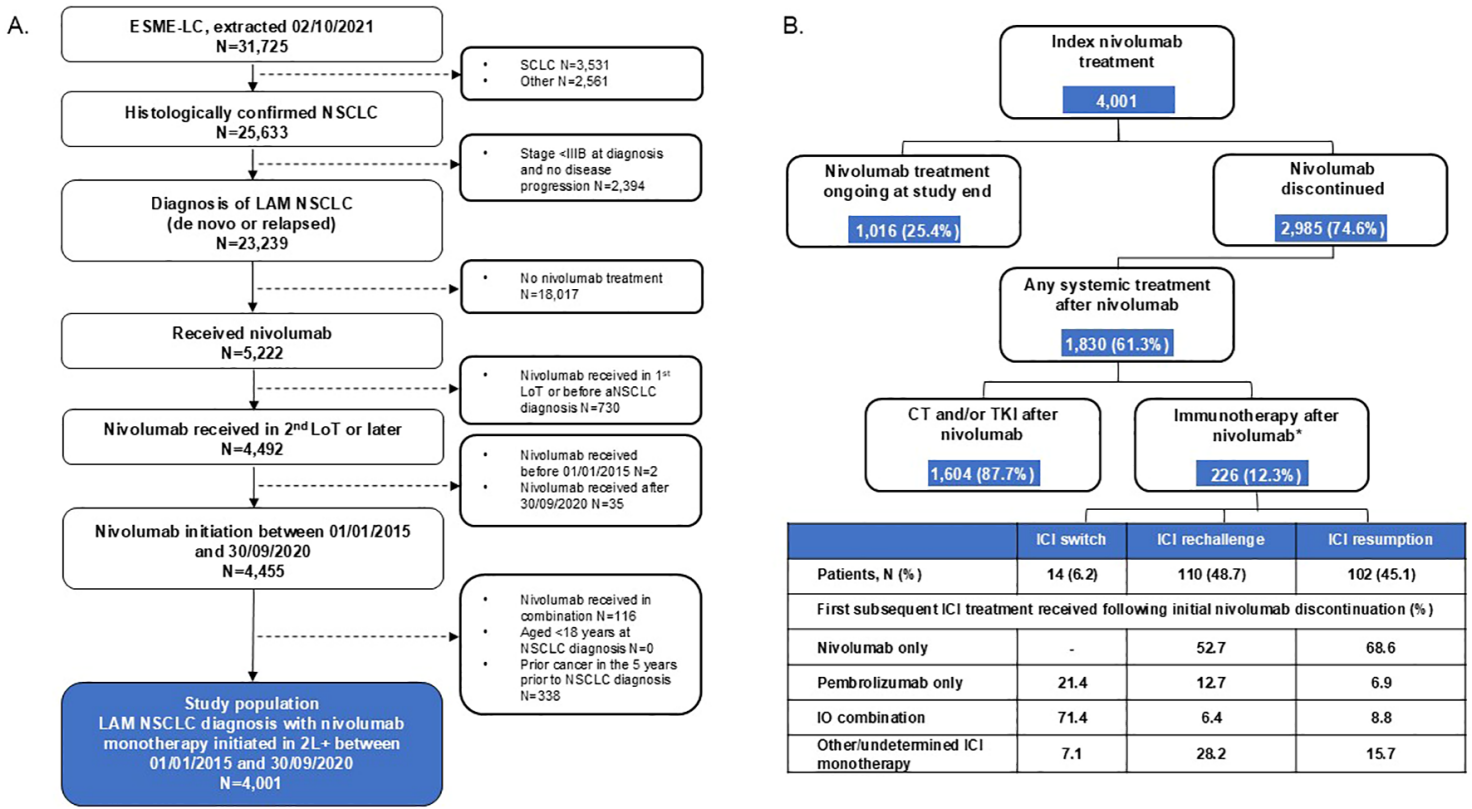
Patient disposition for the overall study cohort of patients with LAM NSCLC who received index nivolumab **(A)** and in patients receiving ICI retreatment or chemotherapy and/or TKI after index nivolumab index discontinuation **(B)**. *Retreatment category for one patient was not available. 2L+, second line or later; aNSCLC, advanced non-small cell lung cancer; CT, chemotherapy; ESME-LC, Epidemiological-Strategy and Medical Economics Lung Cancer; ICI, immune checkpoint inhibitor; IO, immunotherapy; LAM, locally advanced or metastatic; LoT, line of therapy; NSCLC, non-small cell lung cancer; SCLC, small cell lung cancer; TKI, tyrosine kinase inhibitor.

Of a total of 31,725 patients enrolled in the ESME-LC data source, 4,001 patients with stage IIIB–IV NSCLC received index nivolumab monotherapy and were included in this analysis (**Figure 1A**).

Demographics and baseline characteristics for patients receiving index nivolumab are shown in **Tables 1** and **2**. The median age at initiation of index nivolumab was 63.8 years (interquartile range, 57.2–70.2) and similar across all strata split by nivolumab duration; 11.7% of patients in the overall population were aged ≥75 years; however, in the stratum on nivolumab for ≥104 weeks only, 7.4% were aged ≥75 years. More than two-thirds of patients were male (68.4%). Only 6.9% of the patient population were non-smokers; 68.9% of patients were diagnosed with stage IV disease, similar across all strata of nivolumab duration. More than three-quarters of patients (76.5%) had an Eastern Cooperative Oncology Group performance status (ECOG PS) of 0–1 at initiation of nivolumab therapy, with a greater proportion of patients with ECOG PS 0–1 continuing on index nivolumab longer. Two-thirds (67.0%) of patients overall were diagnosed with adenocarcinoma, with the group on nivolumab for ≥104 weeks having a greater proportion of patients with adenocarcinoma (76.4%). A total of 91.7% of patients experienced at least one metastatic site at nivolumab initiation. Bone metastases were most common, reported in 46.6% of patients. Liver metastases were present in 26.0% of patients overall, occurring in 32.0% of patients treated with nivolumab for <13 weeks and 15.4% who received nivolumab for ≥104 weeks; conversely, the proportion of patients with brain metastases was consistent across all the strata defined by the duration of index nivolumab treatment at approximately 40.0%. High blood pressure, chronic obstructive pulmonary disease, and diabetes were the most common comorbidities (66.1%, 32.8%, and 23.2%, respectively). One-third (34.9%) of the population had PD-L1 screening; among them, 37.8% had positive (≥1%) PD-L1 expression. Among those with positive PD-L1 expression, 38.0% had PD-L1 ≥50%. The proportion of patients with PD-L1 positivity increased with the duration of time spent on index nivolumab and was the greatest in those on index nivolumab longest (positive PD-L1 expression in 57.7% of patients compared with 34.2% of patients on index nivolumab for ≥104 weeks and <13 weeks, respectively).

**Table 1:** Demographics of patients with LAM NSCLC who received nivolumab in 2L+ at index date.

	Total	Index nivolumab treatment duration (weeks)					
		<13	13–25	26–38	39–51	52–103	≥104
*Population size, N (%)*	4,001	2,184 (54.6)	752 (18.8)	310 (7.7)	183 (4.6)	356 (8.9)	216 (5.4)
Year of index nivolumab initiation, n (%)							
<2017	1,622 (40.5)	872 (39.9)	330 (43.9)	109 (35.2)	68 (37.2)	122 (34.3)	121 (56.0)
≥2017	2,379 (59.5)	1,312 (60.1)	422 (56.1)	201 (64.8)	115 (62.8)	234 (65.7)	95 (44.0)
Age, years							
Median (Q1–Q3)	63.8 (57.2–70.2)	63.6 (56.7–70.0)	64.7 (58.3–70.5)	64.7 (58.5–70.6)	64.9 (57.8–70.9)	64.5 (59.3–71.0)	60.1 (54.9–68.4)
Age ≥75 years, n (%)	467 (11.7)	254 (11.6)	94 (12.5)	36 (11.6)	14 (7.7)	53 (14.9)	16 (7.4)
Male sex, n (%)	2,735 (68.4)	1,488 (68.1)	515 (68.5)	219 (70.6)	127 (69.4)	242 (68.0)	144 (66.7)
Smoking status, n (%)							
Former smoker	2,187 (56.5)	1,181 (55.8)	421 (58.3)	168 (56.0)	109 (60.9)	198 (57.7)	110 (52.6)
Smoker	1,417 (36.6)	766 (36.2)	252 (34.9)	122 (40.7)	58 (32.4)	127 (37.0)	92 (44.0)
Non-smoker	267 (6.9)	171 (8.1)	49 (6.8)	10 (3.3)	12 (6.7)	18 (5.2)	7 (3.3)
ECOG PS, n (%)	2,192	1,231	405	158	96	181	121
0	413 (18.8)	166 (13.5)	96 (23.7)	45 (28.5)	27 (28.1)	51 (28.2)	28 (23.1)
1	1,265 (57.7)	675 (54.8)	255 (63.0)	86 (54.4)	56 (58.3)	111 (61.3)	82 (67.8)
2+	514 (23.4)	390 (31.7)	54 (13.3)	27 (17.1)	13 (13.5)	19 (10.5)	11 (9.1)
Comorbidities recorded at lung cancer diagnosis							
Medical history available, n	3,808	2077	717	295	178	340	201
Record of comorbidities, n (%)	1,840 (48.3)	958 (46.1)	370 (51.6)	147 (49.8)	93 (52.2)	179 (52.6)	93 (46.3)
High blood pressure, n (%)	1,217 (66.1)	638 (66.6)	231 (62.4)	100 (68.0)	68 (73.1)	115 (64.2)	65 (69.9)
COPD, n (%)	604 (32.8)	304 (31.7)	116 (31.4)	55 (37.4)	35 (37.6)	68 (38.0)	26 (28.0)
Diabetes mellitus, n (%)	426 (23.2)	217 (22.7)	96 (25.9)	33 (22.4)	19 (20.4)	41 (22.9)	20 (21.5)

2L+, second line or later; COPD, chronic obstructive pulmonary disease; ECOG PS, Eastern Cooperative Oncology Group performance status; LAM, locally advanced or metastatic; NSCLC, non-small cell lung cancer; Q, quartile.

**Table 2:** Clinical characteristics of patients with LAM NSCLC who received nivolumab in 2L+ at index date.

	Total	Index nivolumab treatment duration (weeks)					
		<13	13–25	26–38	39–51	52–103	≥104
TNM stage at initial diagnosis, n (%)	3,938	2,154	738	308	181	347	210
I–III	1,223 (31.1)	632 (29.3)	250 (33.9)	112 (36.4)	58 (32.0)	109 (31.4)	62 (29.5)
IV	2,715 (68.9)	1,522 (70.7)	488 (66.1)	196 (63.6)	123 (68.0)	238 (68.6)	148 (70.5)
NSCLC histological subtype, n (%)							
Adenocarcinoma	2,682 (67.0)	1,485 (68.0)	481 (64.0)	196 (63.2)	113 (61.7)	242 (68.0)	165 (76.4)
At least one metastatic site, n (%)	3,670 (91.7)	2,035 (93.2)	682 (90.7)	274 (88.4)	164 (89.6)	314 (88.2)	201 (93.1)
Location of metastases, n (%)							
Bone metastases	1,712 (46.6)	1,076 (52.9)	302 (44.3)	106 (38.7)	64 (39.0)	92 (29.3)	72 (35.8)
Brain metastases	1,432 (39.0)	819 (40.2)	232 (34.0)	93 (33.9)	64 (39.0)	141 (44.9)	83 (41.3)
Contralateral lung metastases	1,373 (37.4)	751 (36.9)	264 (38.7)	106 (38.7)	69 (42.1)	116 (36.9)	67 (33.3)
Liver metastases	953 (26.0)	652 (32.0)	156 (22.9)	45 (16.4)	30 (18.3)	39 (12.4)	31 (15.4)
PD-L1 screening,* n (%)	1,396 (34.9)	784 (35.9)	248 (33.0)	119 (38.4)	70 (38.3)	123 (34.6)	52 (24.1)
PD-L1 screening test result							
Negative, n (%)	828 (59.3)	492 (62.8)	155 (62.5)	65 (54.6)	39 (55.7)	55 (44.7)	22 (42.3)
Positive, n (%)	528 (37.8)	268 (34.2)	88 (35.5)	45 (37.8)	30 (42.9)	67 (54.5)	30 (57.7)
If positive (≥1%), category of tumor-activated cells							
1% to 49% expression, n (%)	295 (62.0)	152 (62.6)	49 (62.8)	22 (53.7)	21 (77.8)	38 (63.3)	13 (48.1)
≥50% expression, n (%)	181 (38.0%)	91 (37.4)	29 (37.2)	19 (46.3)	6 (22.2)	22 (36.7)	14 (51.9)

2L+, second line or later; LAM, locally advanced or metastatic; NSCLC, non-small cell lung cancer; PD-L1, programmed death ligand 1; TNM, tumor, node, metastasis.

*Any time during the course of the disease.

### 3.2 Treatment characteristics

Overall, the median duration of 1L therapy was 3.0 months. The median time between LAM NSCLC diagnosis and index nivolumab initiation was 9.7 months (**Table 3**). The most common 1L therapy was platinum-based chemotherapy, received by 92.8% of patients. A total of 2,442 patients received nivolumab as 2L therapy and 1,559 as 3L+ (**Supplementary Figure S1**). One-third of all patients (33.5%) received two lines of therapy (LoTs), while 28.0% and 38.5% received three and four or later LoTs, respectively, over the entire follow-up period (**Table 3**).

**Table 3:** Treatment characteristics of patients with LAM NSCLC who received nivolumab in 2L+ at index date.

	Total	Index nivolumab treatment duration (weeks)					
		<13	13–25	26–38	39–51	52–103	≥104
Lines of therapy received over the entire follow-up, n (%)							
2	1,339 (33.5)	744 (34.1)	200 (26.6)	85 (27.4)	55 (30.1)	150 (42.1)	105 (48.6)
3	1,120 (28.0)	636 (29.1)	201 (26.7)	92 (29.7)	44 (24.0)	85 (23.9)	62 (28.7)
4+	1,542 (38.5)	804 (29.1)	351 (46.7)	133 (42.9)	84 (45.9)	121 (34.0)	49 (22.7)
Median time (Q1–Q3) between first and second lines of therapy, months	5.9 (3.1–9.8)	5.3 (2.9–8.7)	6.5 (3.4–10.9)	7.1 (3.7-11.2)	6.6 (3.2–10.3)	7.6 (3.9–12.6)	6.6 (3.7–11.6)
Time between LAM NSCLC diagnosis to index nivolumab initiation, median (IQR), months	9.7 (6.3–16.4)	8.8 (5.8–14.2)	10.8 (6.7–18.1)	11 (7.5–17.8)	10.8 (7.0–19.3)	11.5 (7.8–21.1)	11.7 (6.5–19.5)
1L duration, median (IQR), months	3 (2.1–5.1)	2.8 (1.9–4.8)	3.2 (2.1–5.4)	3.5 (2.1–5.8)	3.1 (1.7–5.6)	3.2 (2.1–5.3)	3.3 (2.0–5.7)
Type of 1L treatment received, n (%)							
Platinum CT	3,712 (92.8)	2,012 (92.1)	701 (93.2)	285 (91.9)	171 (93.4)	340 (95.5)	203 (94.0)
Non-platinum CT	146 (3.6)	76 (3.5)	29 (3.9)	17 (5.5)	7 (3.8)	11 (3.1)	6 (2.8)
PKI	94 (2.3)	66 (3.0)	14 (1.9)	4 (1.3)	3 (1.6)	2 (0.6)	5 (2.3)
ICI	23 (0.6)	17 (0.8)	4 (0.5)	0 (0.0)	0 (0.0)	2 (0.9)	0 (0.0)
Other*	26 (0.6)	13 (0.6)	4 (0.5)	4 (1.3)	2 (1.1)	1 (0.3)	2 (0.9)
Nivolumab discontinuation, n (%)	2,985 (74.6)	1,633 (74.8)	613 (81.5)	242 (78.1)	140 (76.5)	240 (67.4)	117 (54.2)
If discontinued, reason (multiple possible)							
Disease progression, n (%)	2,018 (67.6)	1,148 (70.3)	454 (74.1)	160 (66.1)	91 (65.0)	127 (52.9)	38 (32.5)
Doctor’s choice/protocol-driven choice, n (%)	574 (19.2)	280 (17.1)	93 (15.2)	48 (19.8)	25 (17.9)	68 (28.3)	60 (51.3)
Toxicity, n (%)	290 (9.7)	133 (8.1)	55 (9.0)	32 (13.2)	22 (15.7)	33 (13.8)	15 (12.8)
Patient’s choice, n (%)	51 (1.7)	29 (1.8)	7 (1.1)	2 (0.8)	2 (1.4)	7 (2.9)	4 (3.4)
Other, n (%)	220 (7.4)	130 (8.0)	35 (5.7)	14 (5.8)	12 (8.6)	24 (10.0)	5 (4.3)

1L, first line; 2L+, second line or later; ICI, immune checkpoint inhibitor; IQR, interquartile range; LAM, locally advanced or metastatic; NSCLC, non-small cell lung cancer; PKI, protein kinase inhibitor; Q, quartile.

*Patients in clinical trials and those treated with other monoclonal antibodies.

More than one-half (54.6%) of patients included in this analysis remained on index nivolumab for <13 weeks, with 18.8% receiving it for 13–25 weeks, 7.7% for 26–38 weeks, 4.6% for 39–51 weeks, 8.9% for 52–103 weeks, and 5.4% of patients receiving nivolumab for ≥104 weeks. The proportion of patients who received index nivolumab therapy for ≥104 weeks was 56.0% for those starting therapy prior to 2017 and 44.0% for those starting after 2017. Patients with longer index nivolumab durations had fewer LoTs overall (**Table 3**).

#### 3.2.1 Index nivolumab discontinuation

Overall, 74.6% of patients discontinued index nivolumab during follow-up (**Figure 1B**), with the most common reason overall being disease progression (67.6%). The second most common reason for discontinuation was doctor’s choice/protocol-driven choice (19.2%), which became more common in patients with longer index nivolumab treatment duration (**Table 3**).

The median time from index nivolumab discontinuation to end of follow-up was 5.5 (interquartile range, 5.3–5.9) months overall (**Supplementary Table S1**), which increased in those receiving index nivolumab for longer: 3.5 months in those on index nivolumab for <13 weeks to 10.3 months for those completing ≥26 weeks of index nivolumab.

### 3.3 PFS, TTDD, and OS from initiation of index nivolumab treatment

**Figure 2:**
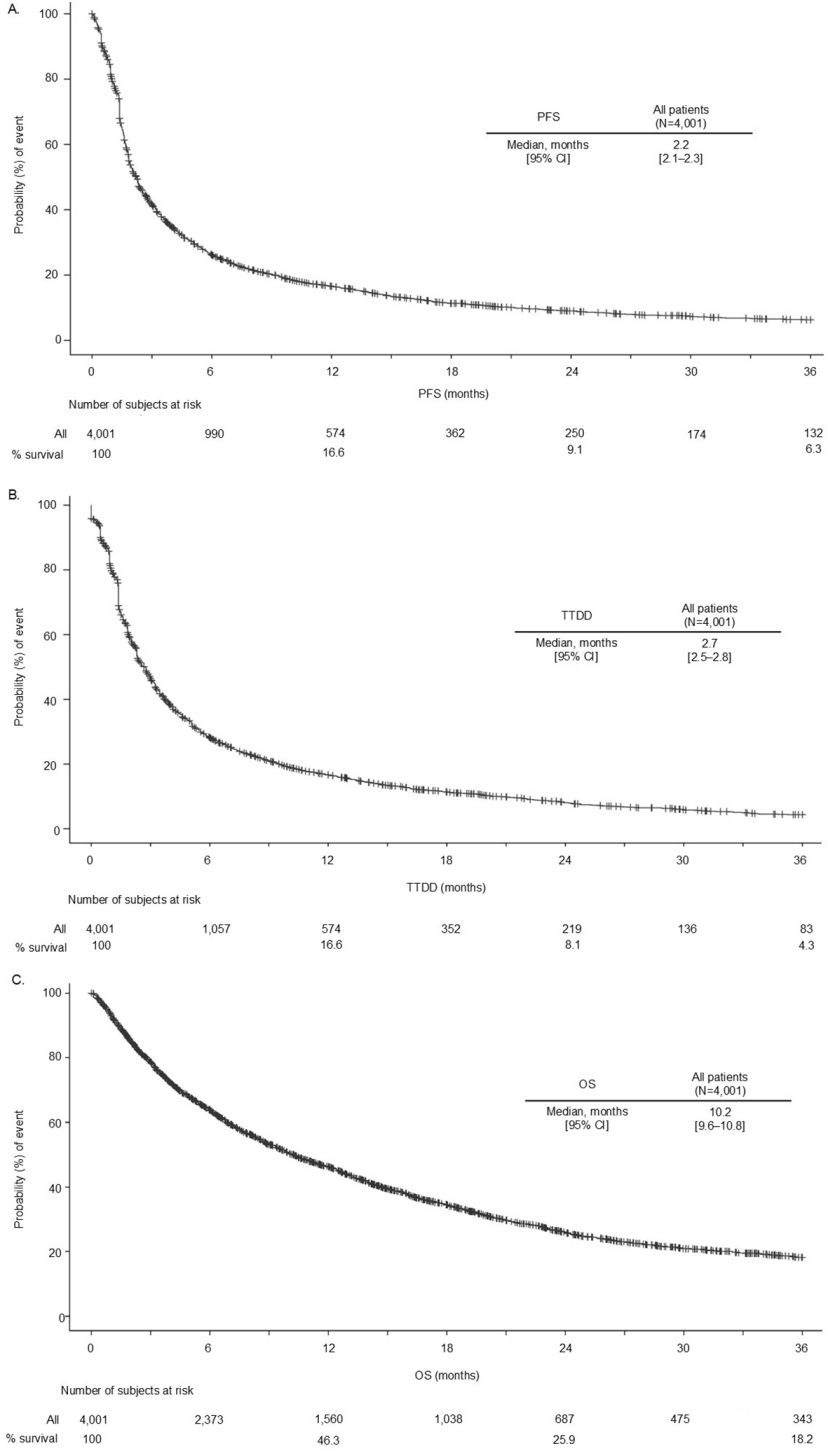
PFS **(A)**, TTDD **(B)**, and OS **(C)** in patients with LAM NSCLC who received index nivolumab. 2L+, second line or later; CI, confidence interval; LAM, locally advanced or metastatic; NSCLC, non-small cell lung cancer; OS, overall survival; PFS, progression-free survival; TTDD, time to treatment discontinuation or death.

The median [95% CI] PFS was 2.2 [2.1–2.3] months (**Figure 2A**); 1-year PFS was 16.6%, and 2-year PFS was 9.1%. The median [95% CI] TTDD was 2.7 [2.5–2.8] months (**Figure 2B**). The median [95% CI] OS was 10.2 [9.6–10.8] months (**Figure 2C**); 1-year OS was 46.3%, and 2-year OS was 25.9%.

There was a trend in median OS and 1-year PFS rates according to PD-L1 status for longer survival outcomes in patients who had PD-L1 expression ≥50%; however, this was not formally tested, and CIs overlapped (**Supplementary Figures S2A**, **S3B**). Additionally, the results for the median PFS suggested the longest duration for patients with 1%–49% expression.

For the landmark analyses at 13 weeks, 26 weeks, 39 weeks, and 52 weeks, stratified by whether patients were continuing index nivolumab treatment or not, subsequent PFS and OS increased markedly with each landmark achieved in patients continuing nivolumab therapy (**Supplementary Figures S3A, B**): the median PFS [95% CI] at 13 weeks was 6.3 [5.3–6.8] months and 15.2 [12.7–18.9] months at 52 weeks; the median OS [95% CI] at 13 weeks was 19.9 [18.4–21.1] months and 40.3 [34.7–45.9] months at 52 weeks.

### 3.4 Retreatment after nivolumab discontinuation

After discontinuing nivolumab, 61.3% (n = 1,830) of patients received further treatment (**Figure 1B**, **Tables 4**, **5**). Of these, most were treated with chemotherapy and/or TKI (87.7%, n = 1,604), whereas 12.3% (n = 226) received further ICI. Among patients who received index nivolumab for a duration of >26 weeks, ICI retreatment rates were higher (15.8% overall) than among patients who used index nivolumab for <13 weeks (3.5% overall) (**Supplementary Table S1**). Of those receiving ICI retreatment following index nivolumab discontinuation, 48.7% (n = 110), 45.1% (n = 102), and 6.2% (n = 14) were in the rechallenge, resumption, and switch categories, respectively (**Table 4**).

**Table 4:** Demographics and clinical characteristics of retreated patients following index nivolumab discontinuation according to their type of retreatment.

	CT and/or TKI subgroup (n = 1,604)	Rechallenge (n = 110)	Resumption (n = 102)	Switch (n = 14)
Median (Q1–Q3) age at nivolumab initiation, years	63.2 (56.6–69.5)	61.5 (54.6–69.0)	66.3 (59.3–72.2)	59.1 (52.9–64.1)
Age ≥75 years at nivolumab initiation, n (%)	146 (9.1)	7 (6.4)	15 (14.7)	1 (7.1)
Male sex, %	66.7	63.6	71.6	71.4
LAM NSCLC diagnosis type, %				
*De novo*	82.2	81.8	68.6	85.7
NSCLC histological subtype, n (%)				
Adenocarcinoma	1,076 (67.1)	75 (68.2)	71 (69.6)	11 (78.6)
PD-L1 screening,* %	34.5	37.3	40.2	64.3
PD-L1 screening test result				
Positive, %	36.3	43.9	53.7	66.7
If positive (≥1%), category of tumor-activated cells				
1% to 49% expression, n (%)	122 (65.6)	9 (60.0)	9 (45.0)	4 (80.0)
≥50% expression, n (%)	64 (34.4)	6 (40.0)	11 (55.0)	1 (20.0)
TNM stage at initial diagnosis, n	1,577	110	100	13
I–III, %	31.8	34.4	38.2	21.4
IV, %	66.6	65.4	59.8	71.4
Not available, %	1.7	0	2	7.1
ECOG performance status at nivolumab initiation, n	901	64	58	7
0, %	24.1	37.5	32.8	28.6
1, %	64.8	54.7	50	57.1
≥2, %	11.1	7.8	17.2	14.3
Location of metastases, %				
Brain	37.3	31.3	30.9	53.8

2L+, second line or later; CT, chemotherapy; ECOG, Eastern Cooperative Oncology Group; LAM, locally advanced or metastatic; NSCLC, non-small cell lung cancer; PD-L1, programmed death ligand 1; Q, quartile; TNM, tumor, node, metastasis; TKI, tyrosine kinase inhibitor.

*Any time during the course of the disease.

**Table 5:** Treatment characteristics of treated patients following index nivolumab discontinuation according to their retreatment type.

	CT and/or TKI (n = 1,604)	Rechallenge (n = 110)	Resumption (n = 102)	Switch (n = 14)
Median follow-up from index nivolumab initiation, months (range)	11.4 (1.0–73.4)	31.9 (7.6–70.4)	29.9 (5.1–73.9)	24.8 (4.4–63.4)
Duration of first LoT for LAM NSCLC, months, median (Q1–Q3)	3.3 (2.1–5.6)	3.3 (2.2–5.9)	3.6 (2.0–5.3)	4.6 (3.5–8.0)
ICI as 1L therapy for LAM NSCLC, n (%)	8 (0.5)	0 (0.0)	1 (1.0)	0 (0.0)
LoT of first nivolumab treatment received, n (%)				
2L	948 (59.1)	72 (65.5)	58 (56.9)	7 (50.0)
3L	433 (27.0)	23 (20.9)	27 (26.5)	5 (35.7)
≥4L	223 (13.9)	15 (13.6)	17 (16.7)	2 (14.3)
Initial nivolumab treatment duration, months, median (Q1–Q3)	2.5 (1.4–5.1)	5.4 (2.7–10.6)	6.9 (3.0–18.2)	7.0 (6.0–16.1)
Reason for discontinuation, n (%)				
Progression	1,350 (84.2)	86 (78.2)	25 (24.5)	11 (78.6)
Toxicity	101 (6.3)	8 (7.3)	23 (22.5)	–
Patient’s choice	3 (0.2)	–	7 (6.9)	–
Doctor’s choice/protocol-driven choice	156 (9.7)	10 (9.1)	43 (42.2)	2 (14.3)
Other	53 (3.3)	7 (6.4)	16 (15.7)	1 (7.1)
Median time from nivolumab discontinuation to retreatment with ICI, months (IQR)	NA	11.5 (7.0–19.5)	5.6 (3.1–10.3)	1.0 (0.9–1.1)
Median ICI retreatment duration, months (IQR)	NA	2.1 (1.1–5.2)	3.3 (1.4–6.5)	3.7 (1.7–5.6)

Follow-up was calculated as follows: (date of last medical information-index date)/30.4375. This is a time interval calculation; no censoring rule was applied.

1L, first line; 2L, second line; 2L+, second line or later; 3L, third line; ≥4L, fourth line or later; CT, chemotherapy; ICI, immune checkpoint inhibitor; IQR, interquartile range; LAM, locally advanced or metastatic; LoT, line of therapy; NA, not available; NSCLC, non-small cell lung cancer; Q, quartile; TKI, tyrosine kinase inhibitor.

Some of the main demographic characteristics varied across the retreatment subgroups. The median age ranged from 59.1 to 66.3 years in the switch and resumption subgroups, respectively, with the proportion of >75-year-old patients ranging from 6.4% in the rechallenge subgroup to 14.7% in the resumption subgroup (**Table 4**). The proportion of male patients ranged from 63.6% in the rechallenge subgroup to 71.6% in the resumption subgroup, and ECOG PS 0–1 ranged from 82.8% to 92.2% in the resumption and rechallenge subgroups, respectively, compared with 76.6% of the overall index nivolumab population. Brain metastasis rates ranged from 30.9% in the resumption subgroup to 53.7% in the switch subgroup, compared with 39.0% in the index nivolumab population. The median duration of index nivolumab treatment ranged from 2.5 months in the chemotherapy and/or TKI group to 7.0 months in the switch group (**Table 5**).

Of those patients receiving retreatment with ICI therapy, 25.7% were on index nivolumab for <13 weeks, 22.6% for 13–25 weeks, and 51.8% for ≥26 weeks. In those who retreated with chemotherapy and/or TKI, over one-half (55.2%) received index nivolumab for <13 weeks. The majority of patients who were rechallenged or switched ICI therapy discontinued index nivolumab due to disease progression (78.2% and 78.6%, respectively), similar to those who retreated with chemotherapy and/or TKI, while for those in the resumption group, the most common reason for index nivolumab discontinuation was doctor’s choice/protocol-driven choice (42.2%). A larger proportion of patients in the ICI resumption subgroup discontinued index nivolumab owing to toxicity (22.5%), whereas, in the rechallenge subgroup, toxicity was the reason for discontinuation in only 7.3% of patients.

The median follow-up from index nivolumab initiation was longer for patients retreated with an ICI, in particular, those rechallenged (31.9 months) or resuming an ICI (29.9 months) compared with those who received chemotherapy and/or TKI (11.4 months) (**Table 5**).

**Figure 3:**
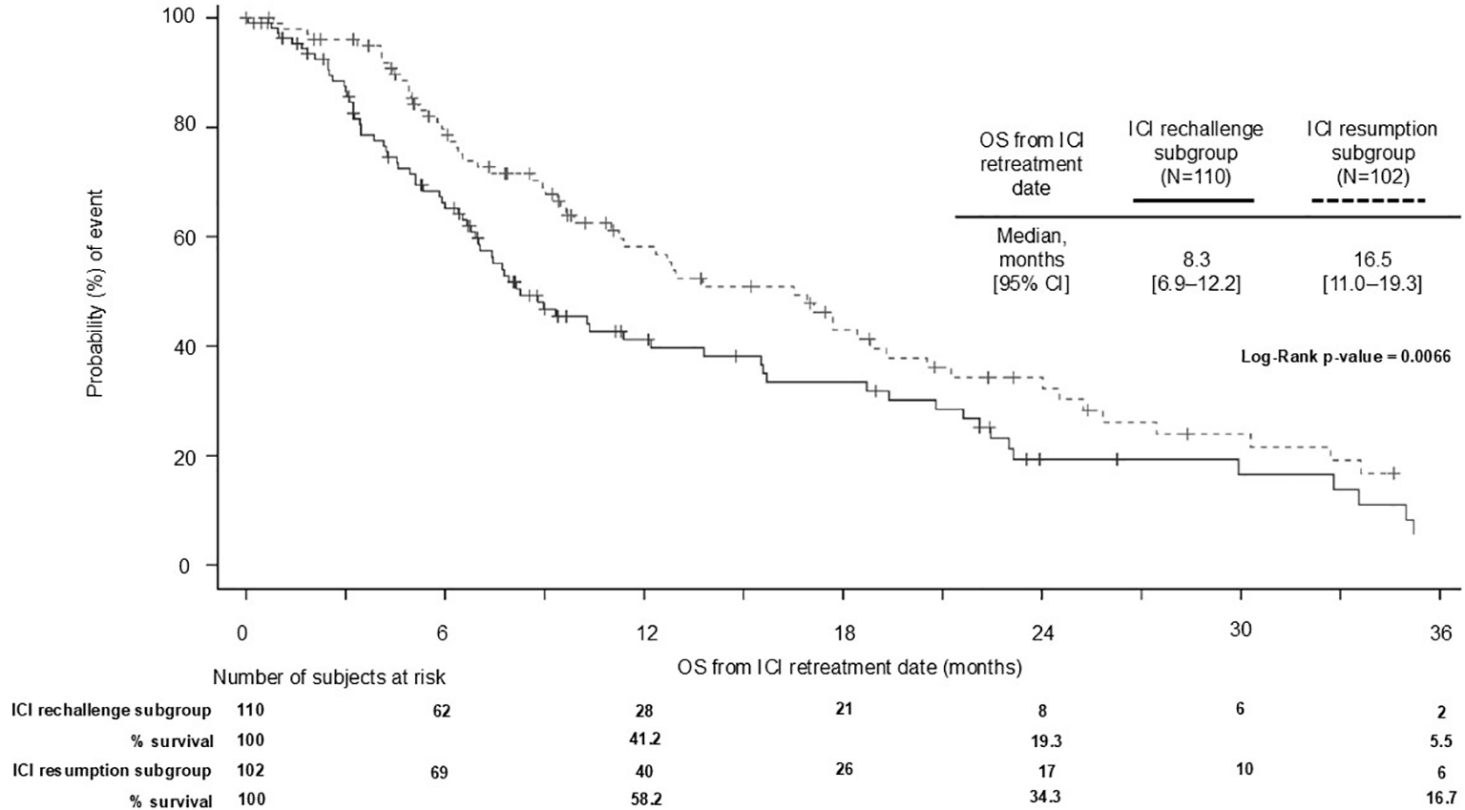
OS from retreatment with ICI initiation in patients with ICI resumption and ICI rechallenge following index nivolumab discontinuation. 2L+, second line or later; CI, confidence interval; ICI, immune checkpoint inhibitor; OS, overall survival.

Patients who received ICI retreatment had a longer median OS if they were in the resumption group rather than the rechallenge group (**Figure 3**); the median [95% CI] OS from the retreatment start date was 16.5 [11.0–19.3] months versus 8.3 [6.9–12.2] months for patients who resumed or were rechallenged, respectively. The 1- and 2-year OS rates from the retreatment date of those in the resumption subgroup were 13.4% and 7.9%, respectively, compared with those in the rechallenge subgroup, whose 1- and 2-year OS rates were 9.5% and 4.4%, respectively.

### 3.5 Sensitivity analysis assessing patients who received index nivolumab in 2L only

In a sensitivity analysis of patients who received nivolumab in 2L only (n = 2,442; not including patients receiving nivolumab in 3L+), baseline demographics and patient characteristics were similar to those who received nivolumab in 2L+ (**Supplementary Table S2**). Treatment characteristics were also similar; notably, 20.4% of 2L-only patients received ≥4 LoTs during follow-up (**Supplementary Table S2**), compared with 38.5% of patients when grouped as index nivolumab (**Table 3**), while retreatment patterns were similar between both 2L-only (**Supplementary Table S3**) and 2L+ patients (**Supplementary Table S1**). PFS and OS outcomes for those treated with nivolumab in 2L only were also similar to those observed when including all patients receiving index nivolumab therapy (**Supplementary Table S4**).

## 4 Discussion

As the treatment landscape for patients with LAM NSCLC evolves and therapeutic options increase, an understanding of real-world clinical and treatment characteristics, along with survival outcomes, can assist in bridging the knowledge gap between randomized controlled trials and real-world therapy use.

This large study, capturing patient data over a 5-year inclusion period during which time ICI use in the treatment of patients with LAM NSCLC has evolved rapidly, has provided real-world data from 4,001 patients within the French ESME-LC database, as part of the I-O Optimise program. This has allowed the evaluation of real-world patient characteristics and treatment patterns for patients receiving index nivolumab therapy for LAM NSCLC.

OS in this real-world analysis was similar to those in the pivotal Phase 3 studies of nivolumab. The median OS was 10.2 months in this analysis and 12.2 and 9.2 months in CheckMate 057 and CheckMate 017, respectively (15, 16). The estimated 1- and 2-year OS rates were 46.3% and 25.9%, respectively, in our analysis, similar to 48.0% OS at 1 year and 26.9% OS at 2 years in the pooled CheckMate 017 and CheckMate 057 analysis (10). Our study reports similar OS rates to the real-world UNIVOC study with 49.2% at 1 year and 29.8% at 2 years (24). The real-world results presented in this study are encouraging, particularly considering the higher incidence of patients with brain metastases in our cohort (39.0%) compared with CheckMate 057 and CheckMate 017 (12.0% and 7.0%, respectively) (15, 16). The UNIVOC (24) and EVIDENS (25) real-world analyses previously demonstrated a shorter median OS in patients with brain metastases than without, although the correlation between brain metastases and survival outcomes in patients treated with immunotherapy remains inconclusive (26). Additionally, 23.4% of patients in our study had an ECOG PS of ≥2, whereas patients included in CheckMate 057 and CheckMate 017 were required to have an ECOG PS of ≤1 for study inclusion.

Estimated PFS rates at 1 year were also similar between our analysis (16.6%) and the CheckMate 057 and CheckMate 017 trials at 19.0% and 21.0%, respectively (15, 16).

Within the population of this study, approximately two-thirds of patients did not receive a PD-L1 screening test at any time during the course of their disease. In those who did, there was a suggestive trend for median OS to be longer in patients expressing higher levels of PD-L1; however, there was a borderline statistically significant difference in PFS curves according to the duration of index nivolumab treatment (p = 0.046). OS and PFS rates at 1 and 2 years consistently showed improvement as PD-L1 expression levels increased. A recent meta-analysis evaluating outcomes from trials in patients with LAM NSCLC and ECOG PS ≤ 1, pretreated with an ICI, reported similar PFS rates to those we report here at 1 and 2 years of 13.2% and 7.7% in those with PD-L1 <1%, 21.9% and 7.4% for patients with PD-L1 expression 1–49%, and 27.5% and 21.3% for those with PD-L1 ≥50%. OS rates at 2 years reported in this meta-analysis of 25.4%, 28.3%, and 40.5% in patients with PD-L1 levels <1%, 1–49%, and ≥50%, respectively, while greater than those seen in our analysis, followed a similar trend as PD-L1 levels increased (27).

Landmark analyses in nivolumab continuers demonstrated a substantial increase in the median OS and PFS over time across specific timepoints of 13 weeks, 26 weeks, 39 weeks, and 52 weeks after index nivolumab initiation. These patients were also more likely to be retreated and have longer survival from retreatment.

Before commencing index nivolumab monotherapy, platinum-based chemotherapy was the most frequently used 1L therapy for LAM NSCLC. Despite pembrolizumab reimbursement in 2017 as 1L monotherapy for patients with LAM NSCLC and PD-L1 ≥50% in France (9), only 0.6% of our study population was treated with an ICI (other than nivolumab) as 1L therapy prior to index nivolumab, yet almost 60% of our cohort commenced index nivolumab in or after 2017. One explanation for this is that patients were not given 1L ICI because of comorbidity and that nivolumab was given as 2L instead, suggesting the patients in the 1L ICI era were different with respect to outlook. Compared with the UNIVOC study and an Italian real-world study, our current study had a much higher proportion of patients with brain metastases than those real-world studies (39%, 17%, and 15%, respectively), and chronic obstructive pulmonary disease recorded as a comorbidity (32.8% in our study vs. 12.9% in UNIVOC) (18, 28). Although neither of these factors alone may prohibit the commencement of 1L ICI therapy, their impact on ECOG PS and associated concomitant drug therapy (steroids, recurrent antibiotics, etc.) may prevent patients from accessing ICIs as 1L monotherapy. Additionally, it may reflect the duration of time between regulatory and reimbursement approval, and actual uptake of 1L ICI monotherapy usage, suggesting a slow uptake of pembrolizumab monotherapy in France. From the data on 5,222 patients within the ESME-LC database diagnosed with LAM NSCLC and in receipt of nivolumab therapy, 14.0% received this as a 1L of therapy or preceding LAM NSCLC, necessitating their exclusion from the study cohort. It is thought that this is related to patients having early access to nivolumab through the early access Autorisation Temporaire d’Utilisation (Temporary Authorization for Use) program.

The median duration of treatment with index nivolumab for the overall study population was 2.5 months, similar to real-world results observed in UNIVOC (18), which reported a median treatment duration of 2.8 months. However, it was lower than that observed in Italy, where the median time to discontinuation of nivolumab therapy in 2017 was 4.2 months (28). Current European Society for Medical Oncology guidelines advise cessation of PD-L1 therapies upon disease progression (3). Two-thirds of patients in this study discontinued index nivolumab because of disease progression. More than one-half (56.9%) of these patients were on nivolumab for <13 weeks, which is consistent with the rates of primary resistance reported in the literature (29).

In contrast to our study, the real-world UNIVOC study analyzed data from 10,452 patients treated with 2L+ nivolumab therapy in France between 2015 and 2016 and reported much higher retreatment rates (29.6%) with ICIs in France (18). The lower retreatment rates we report highlight how uncommon it is to “rechallenge” patients with nivolumab after they first received an ICI in 2L+. Given the emergence of ICI in 1L treatment, some of the patients treated in 2L+ in our study (in the most recent years) are patients who probably did not receive ICI in 1L, for various reasons as mentioned above (e.g., corticosteroids, temporary contraindication, and oncogenic mutations), and who are possibly more at risk of having an ineffective 2L ICI. The UNIVOC study was conducted during a period when ICI could only be initiated as a 2L therapy (lung cancer diagnosis between 2011 and 2016 and nivolumab treatment in 2015–2016), hence an increased possibility of rechallenge after the first effective ICI sequence. Despite this, evidence is emerging that patients with NSCLC who develop resistance to PD-L1 inhibitor therapies can safely undergo retreatment with ICIs (30).

With the evolution of ICI therapy as a 1L treatment option for patients with LAM NSCLC, retreatment of patients with ICIs is currently an area of great interest. Although not fully understood, the dynamic nature of the immune response can be reinvigorated when ICI treatment is re-established following prior use. Despite evidence suggesting that generalized retreatment of patients with progressive LAM NSCLC with ICI therapies is ineffective, data are emerging proposing that retreatment with ICI therapy following discontinuation for particular subgroups of patients can be beneficial (28, 29). The identification of these individuals is, however, yet to be determined.

Within our study in the resumption group following index nivolumab discontinuation, the median OS from retreatment was shown to increase as the index duration of nivolumab increased (9.4 months [95% CI, 6.3–21.2] with <13 weeks’ nivolumab index therapy to 16.9 [95% CI, 9.9–24.0] months with ≥26 weeks’ index nivolumab therapy duration), aligning with the results observed within the Giaj Levra et al. UNIVOC 2020 cohort (18). This shows that those patients who do well with index ICI therapy with discontinuation due to protocol restrictions, as opposed to primary resistance and disease progression, fare better upon retreatment. Giaj Levra et al. suggested that this may be related to a progressive consolidation of an immune memory during the first treatment course (18).

Our analysis demonstrated that patients undergoing ICI resumption had a longer OS from retreatment than those in the rechallenge subgroup, whereas the UNIVOC study found a greater median OS in those patients being rechallenged with ICI retreatment versus those in the resumption subgroup (18). The variation in these results could possibly be explained by the small numbers of patients in the retreatment groups in our study or by the time periods studied. As ICI therapies are increasingly incorporated as 1L options in LAM NSCLC, the accurate identification of the patients most likely to benefit from retreatment, when inevitable disease progression occurs, becomes more important.

### 4.1 Strengths and limitations

The ESME-LC research program is a comprehensive academic initiative that centralizes real-world data from multiple French cancer centers. Through aggregation of information from various sources, comprehensive patient treatment and outcome data covering a broad patient population are compiled.

The large patient population we report here is derived from the ESME-LC real-world database, which is based on several French cancer centers and is representative of patients with lung cancer in France. This strengthens this analysis; however, there are several limitations that should be acknowledged. First, the study population was derived from a single country and may not be fully representative of the broader European population of patients with NSCLC. Second, the retrospective design may limit this descriptive study interpretation and does not allow control of confounding factors that could have influenced outcome or retreatment rate and modalities. In particular, missing clinical data (for example, a high proportion of missing ECOG data) or absence of treatment-related data, such as the reason for index nivolumab treatment cessation, could potentially have been of interest to contextualize our results. The evolution of the treatment landscape following the approval of pembrolizumab plus chemotherapy as a 1L treatment option for patients with metastatic NSCLC in September 2018 has led to a significant change in therapeutic practice, which may have the potential to affect the uptake of nivolumab in 2L+ and retreatment with ICIs. Data for the years 2020 and 2021 are also likely to be affected by the COVID-19 pandemic. Although the data for these periods are described, it is difficult to determine the extent to which the treatment patterns observed during these years are influenced. Finally, the small number of patients undergoing retreatment can potentially limit the interpretation of these results.

### 4.2 Conclusion

This real-world study describes the clinical experience of patients in France with LAM NSCLC across a 5-year period during which significant evolution of the therapeutic landscape occurred. It has demonstrated that a low proportion of patients receive retreatment with ICI therapy despite increasing evidence to suggest potential benefits of ICI retreatment. It has provided further evidence in support of improved survival outcomes in patients undergoing longer initial ICI treatment and contributes to identifying the most appropriate patients within a large heterogeneous group for whom retreatment would be considered successful by incorporating the reason for initial ICI discontinuation. Larger studies focusing on retreatment with patient numbers that allow for stratification by PD-L1 expression and consideration of the choice of ICI retreatment therapy in relation to that given initially would be invaluable for further investigating nivolumab use and retreatment in the real world.

## Data Availability

The original contributions presented in the study are included in the article/**Supplementary Material**. Further inquiries can be directed to the corresponding author.

## References

[1] GLOBOCAN. Global Cancer Observatory (2020) (Accessed January 23, 2024).

[2] Debieuvre D Molinier O Falchero L Locher C Templement-Grangerat D Meyer N. Lung cancer trends and tumor characteristic changes over 20 years (2000-2020): Results of three French consecutive nationwide prospective cohorts' studies. Lancet Reg Health Eur. (2022) 22:100492. doi: 10.1016/j.lanepe.2022.100492 36108315 PMC9445429

[3] European Society for Medical Oncology, ESMO. Guidelines for Metastatic NSCLC (2020). Available online at: https://interactiveguidelines.esmo.org/esmo-web-app/toc/index.php?subjectAreaID=1&loadPdf=1 (Accessed January 23, 2024).

[4] Li S de Camargo Correia GS Wang J Manochakian R Zhao Y Lou Y. Emerging targeted therapies in advanced non-small-cell lung cancer. Cancers (Basel). (2023) 15:2899. doi: 10.3390/cancers15112899 37296863 PMC10251928

[5] Siringo M Baena J Bote de Cabo H Torres-Jiménez J Zurera M Zugazagoitia J. Future perspectives in the second line therapeutic setting for non-oncogene addicted non-small-cell lung cancer. Cancers (Basel). (2023) 15:5505. doi: 10.3390/cancers15235505 38067208 PMC10705719

[6] Baxevanos P Mountzios G. Novel chemotherapy regimens for advanced lung cancer: have we reached a plateau? Ann Transl Med. (2018) 6:139. doi: 10.21037/atm.2018.04.04 29862228 PMC5952027

[7] Tang S Qin C Hu H Liu T He Y Guo H. Immune checkpoint inhibitors in non-small cell lung cancer: progress, challenges, and prospects. Cells. (2022) 11:320. doi: 10.3390/cells11030320 35159131 PMC8834198

[8] European Medicines Agency. Opdivo European Public Assessment Report . Available online at: https://www.ema.europa.eu/en/medicines/human/EPAR/opdivo (Accessed January 23, 2024).

[9] Chouaid C Thomas M Debieuvre D Durand-Zaleski I Zacharias S Bosquet L. Effectiveness of nivolumab in second-line and later in patients with advanced non-small cell lung cancer in real-life practice in France and Germany: analysis of the ESME-AMLC and CRISP cohorts. Cancers (Basel). (2022) 14:6148. doi: 10.3390/cancers14246148 36551632 PMC9776880

[10] Borghaei H Gettinger S Vokes EE Chow LQM Burgio MA de Castro Carpeno J. Five-year outcomes from the randomized, phase III trials CheckMate 017 and 057: nivolumab versus docetaxel in previously treated non-small-cell lung cancer. J Clin Oncol. (2021) 39:723–33. doi: 10.1200/JCO.20.01605 PMC8078445 33449799

[11] European Medicines Agency. Keytruda European Public Assessment Report. Available online at: https://www.ema.europa.eu/en/medicines/human/EPAR/keytruda (Accessed January 23, 2024).

[12] Vellanki PJ Mulkey F Jaigirdar AA Rodriguez L Wang Y Xu Y. FDA approval summary: nivolumab with ipilimumab and chemotherapy for metastatic non-small cell lung cancer, a collaborative project orbis review. Clin Cancer Res. (2021) 27:3522–7. doi: 10.1158/1078-0432 PMC8254731 33632925

[13] Paz-Ares L Ciuleanu TE Cobo M Schenker M Zurawski B Menezes J. First-line nivolumab plus ipilimumab combined with two cycles of chemotherapy in patients with non-small-cell lung cancer (CheckMate 9LA): an international, randomised, open-label, phase 3 trial. Lancet Oncol. (2021) 22:198–211. doi: 10.1016/S1470-2045(20)30641-0 33476593

[14] Bailey H Lee A Eccles L Yuan Y Burlison H Forshaw C. Treatment patterns and outcomes of patients with metastatic non-small cell lung cancer in five European countries: a real-world evidence survey. BMC Cancer. (2023) 23:603. doi: 10.1186/s12885-023-11074-z 37386452 PMC10311888

[15] Borghaei H Paz-Ares L Horn L Spigel DR Steins M Ready NE. Nivolumab versus docetaxel in advanced nonsquamous non-small-cell lung cancer. N Engl J Med. (2015) 373:1627–39. doi: 10.1056/NEJMoa1507643 PMC5705936 26412456

[16] Brahmer J Reckamp KL Baas P Crinò L Eberhardt WE Poddubskaya E. Nivolumab versus docetaxel in advanced squamous-cell non-small-cell lung cancer. N Engl J Med. (2015) 373:123–35. doi: 10.1056/NEJMoa1504627 PMC4681400 26028407

[17] Yang K Li J Sun Z Zhao L Bai C. Retreatment with immune checkpoint inhibitors in solid tumors: a systematic review. Ther Adv Med Oncol. (2020) 12:1758835920975353. doi: 10.1177/1758835920975353 33294036 PMC7705192

[18] Giaj Levra M Cotté FE Corre R Calvet C Gaudin AF Penrod JR. Immunotherapy rechallenge after nivolumab treatment in advanced non-small cell lung cancer in the real-world setting: A national data base analysis. Lung Cancer. (2020) 140:99–106. doi: 10.1016/j.lungcan.2019.12.017 31911324

[19] Ekman S Griesinger F Baas P Chao D Chouaid C O'Donnell JC. I-O Optimise: a novel multinational real-world research platform in thoracic malignancies. Future Oncol. (2019) 15:1551–63. doi: 10.2217/fon-2019-0025 30852916

[20] Soares M Antunes L Redondo P Borges M Hermans R Patel D. Real-world treatment patterns and survival outcomes for advanced non-small cell lung cancer in the pre-immunotherapy era in Portugal: a retrospective analysis from the I-O Optimise initiative. BMC Pulm Med. (2020) 20:240. doi: 10.1186/s12890-020-01270-z 32912174 PMC7488009

[21] Ekman S Horvat P Rosenlund M Kejs AM Patel D Juarez-Garcia A. Epidemiology and survival outcomes for patients with NSCLC in Scandinavia in the preimmunotherapy era: a SCAN-LEAF retrospective analysis from the I-O Optimise initiative. JTO Clin Res Rep. (2021) 2:100165. doi: 10.1016/j.jtocrr.2021.100165 34590017 PMC8474201

[22] Edge SB Compton CC. The American Joint Committee on Cancer: the 7th edition of the AJCC cancer staging manual and the future of TNM. Ann Surg Oncol. (2010) 17:1471–4. doi: 10.1245/s10434-010-0985-4 20180029

[23] Amin MB Greene FL Edge SB Compton CC Gershenwald JE Brookland RK. The Eighth Edition AJCC Cancer Staging Manual: Continuing to build a bridge from a population-based to a more "personalized" approach to cancer staging. CA Cancer J Clin. (2017) 67:93–9. doi: 10.3322/caac.21388 28094848

[24] Assié JB Corre R Levra MG Calvet CY Gaudin AF Grumberg V. Nivolumab treatment in advanced non-small cell lung cancer: real-world long-term outcomes within overall and special populations (the UNIVOC study). Ther Adv Med Oncol. (2020) 12:1758835920967237. doi: 10.1177/1758835920967237 33403011 PMC7745546

[25] Barlesi F Dixmier A Debieuvre D Raspaud C Auliac JB Benoit N. Effectiveness and safety of nivolumab in the treatment of lung cancer patients in France: preliminary results from the real-world EVIDENS study. Oncoimmunology. (2020) 9:1744898. doi: 10.1080/2162402X.2020.1744898 33457089 PMC7790497

[26] Hu H Xu ZY Zhu Q Liu X Jiang SC Zheng JH. Brain metastases status and immunotherapy efficacy in advanced lung cancer: A systematic review and meta-analysis. Front Immunol. (2021) 12:669398. doi: 10.3389/fimmu.2021.669398 34335570 PMC8316922

[27] Man J Millican J Mulvey A Gebski V Hui R. Response rate and survival at key timepoints with PD-1 blockade vs chemotherapy in PD-L1 subgroups: meta-analysis of metastatic NSCLC trials. JNCI Cancer Spectr. (2021) 5:pkab012. doi: 10.1093/jncics/pkab012 34084999 PMC8160531

[28] Pasello G Lorenzi M Calvetti L Oliani C Pavan A Favaretto A. Multicenter real-world study on effectiveness and early discontinuation predictors in patients with non-small cell lung cancer receiving nivolumab. Oncologist. (2022) 27:e484–93. doi: 10.1093/oncolo/oyac051 PMC9177098 35429394

[29] Huang Y Zhao JJ Soon YY Kee A Tay SH Aminkeng F. Factors predictive of primary resistance to immune checkpoint inhibitors in patients with advanced non-small cell lung cancer. Cancers (Basel). (2023) 15:2733. doi: 10.3390/cancers15102733 37345072 PMC10216169

[30] Cai Z Zhan P Song Y Liu H Lv T. Safety and efficacy of retreatment with immune checkpoint inhibitors in non-small cell lung cancer: a systematic review and meta-analysis. Transl Lung Cancer Res. (2022) 11:1555–66. doi: 10.21037/tlcr-22-140 PMC9459604 36090645

