# Supplementary material for: Real-world treatment and retreatment patterns and outcomes in patients with advanced or metastatic non-small cell lung cancer following nivolumab monotherapy in second line or later in France: an I-O Optimise analysis
Source: Front Oncol. 2025 Feb 20;15:1526931. doi: 10.3389/fonc.2025.1526931 (PMC11883363; doi:10.3389/fonc.2025.1526931)
Supplement: Supplementary file 1 [file DataSheet1.docx]

**Supplementary appendix**

**Supplementary Figure 1.** Flow chart of included patients with LAM NSCLC who received index nivolumab as 2L (sensitivity analysis)


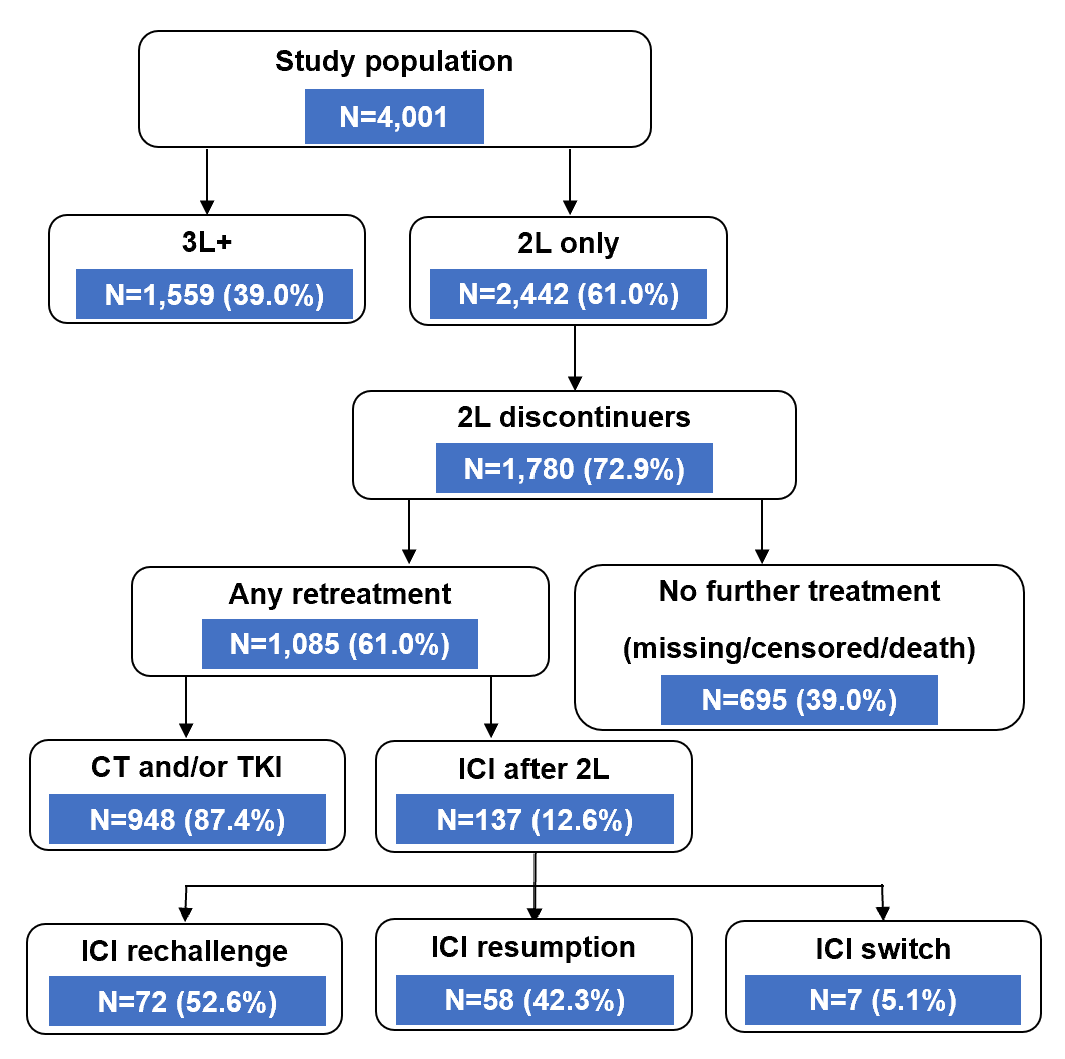


2L, second line; 3L+, third line or later; CT, chemotherapy; ICI, immune checkpoint inhibitor; LAM, locally advanced/metastatic; NSCLC, non-small cell lung cancer; TKI, tyrosine kinase inhibitor.

**Supplementary Figure 2.** PFS (A) and OS (B) Kaplan–Meier curves by PD-L1 expression level in patients with LAM NSCLC who received index nivolumab


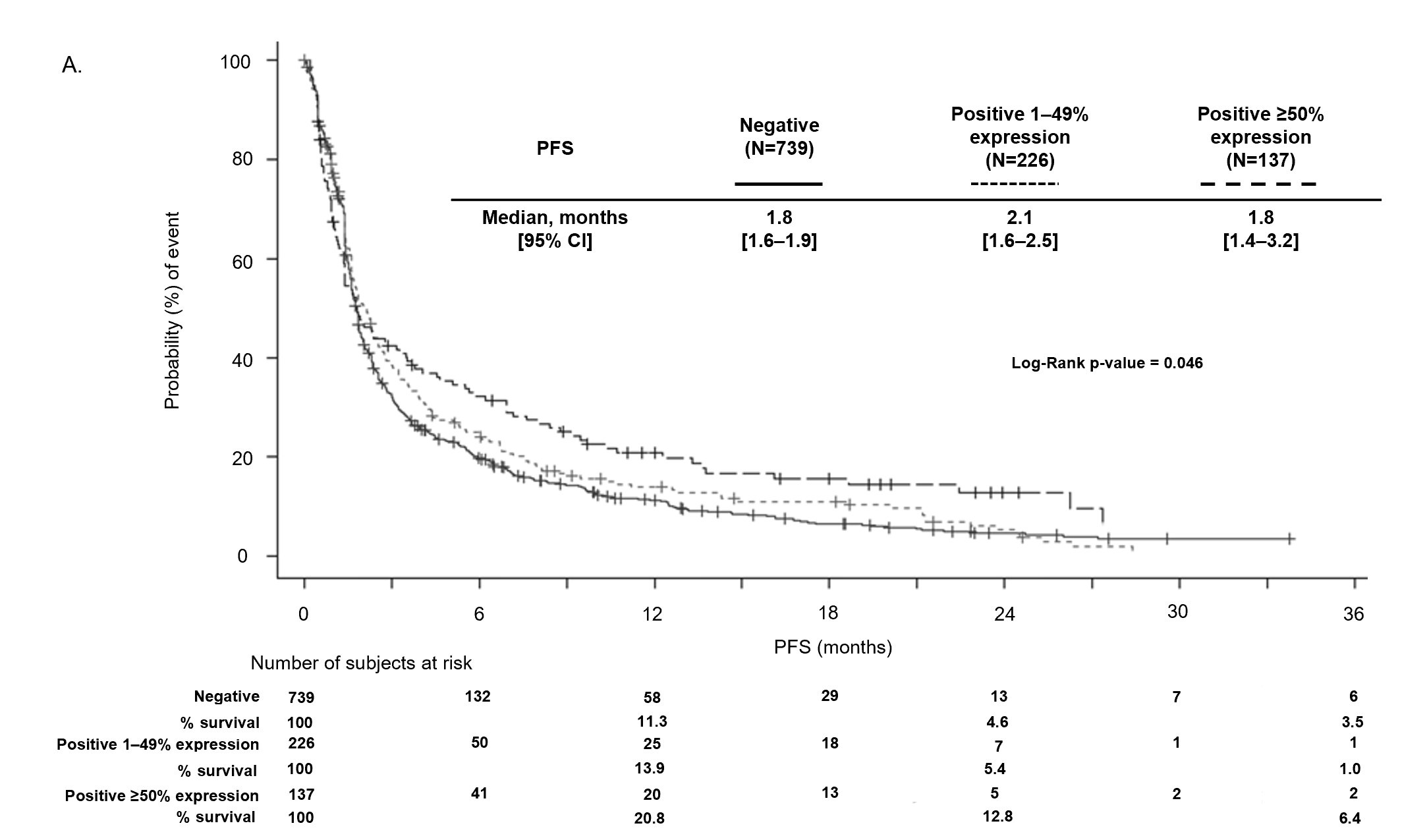


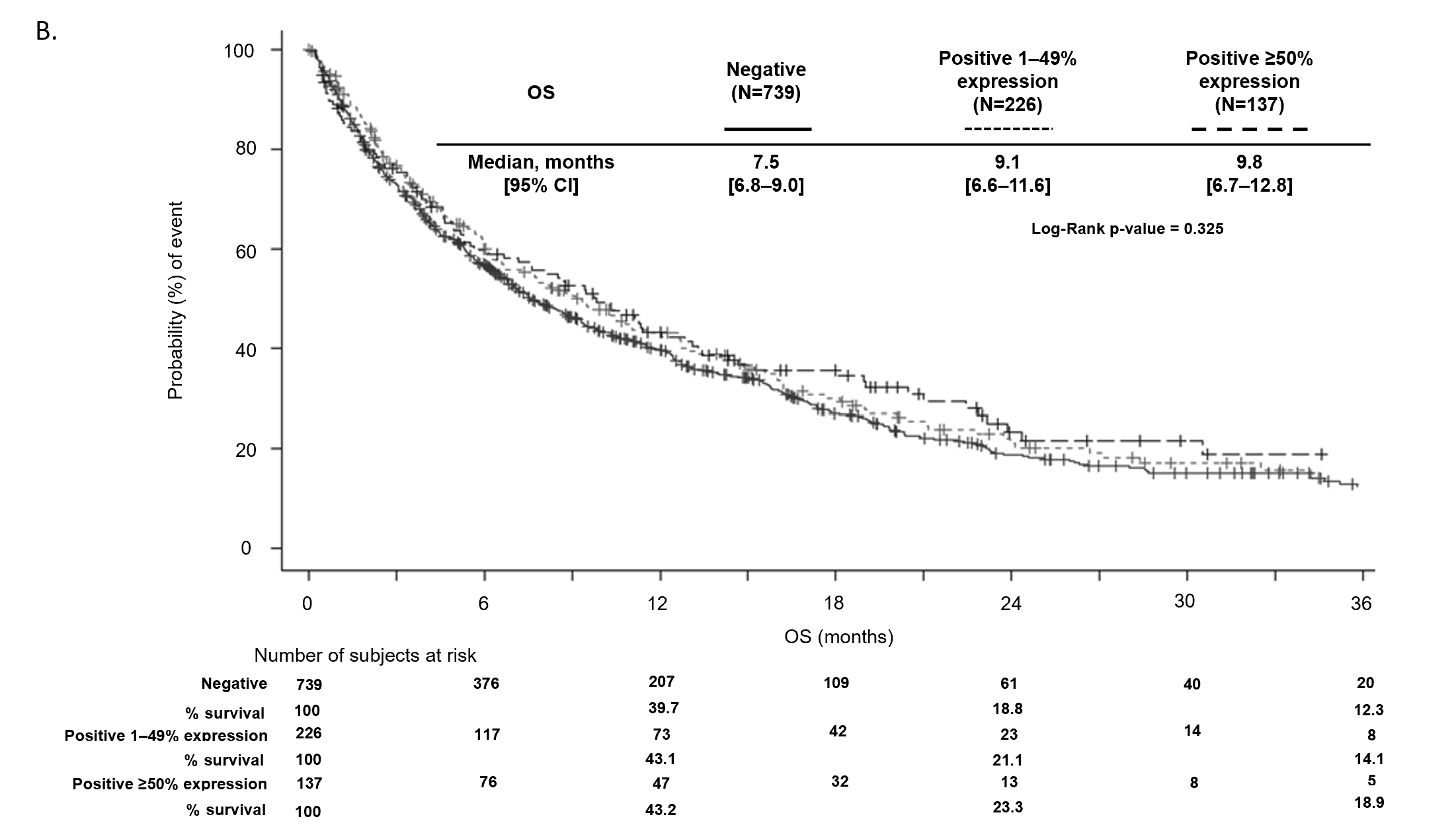


2L+, second line or later; CI, confidence interval; LAM, locally advanced/metastatic; NSCLC, non-small cell lung cancer; OS, overall survival; PD-L1, programmed death ligand 1; PFS, progression-free survival.

**Supplementary Figure 3.** Landmark analysis of PFS (A) and OS (B) in patients with LAM NSCLC who received index nivolumab, by nivolumab treatment status (continued/discontinued)


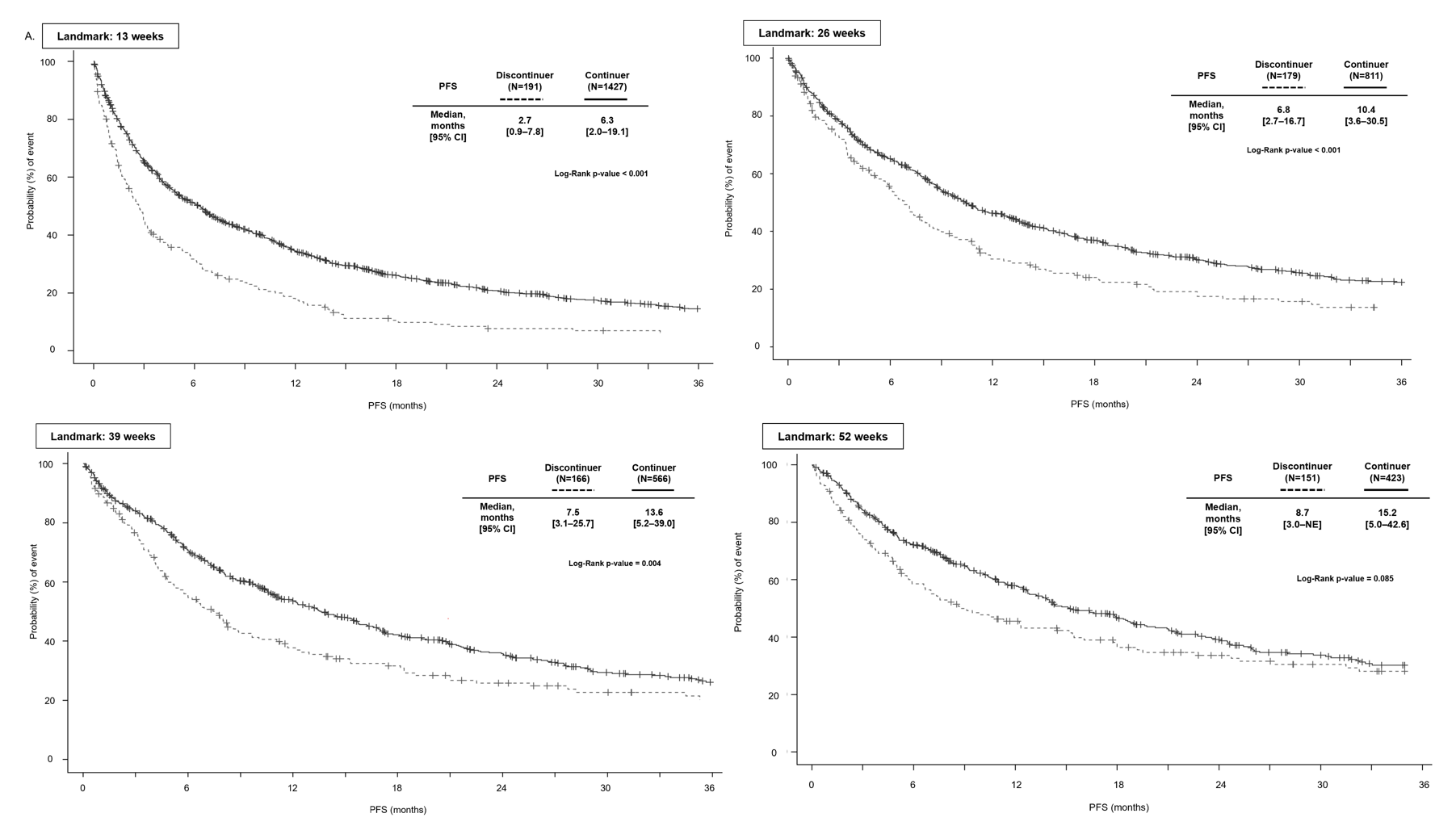


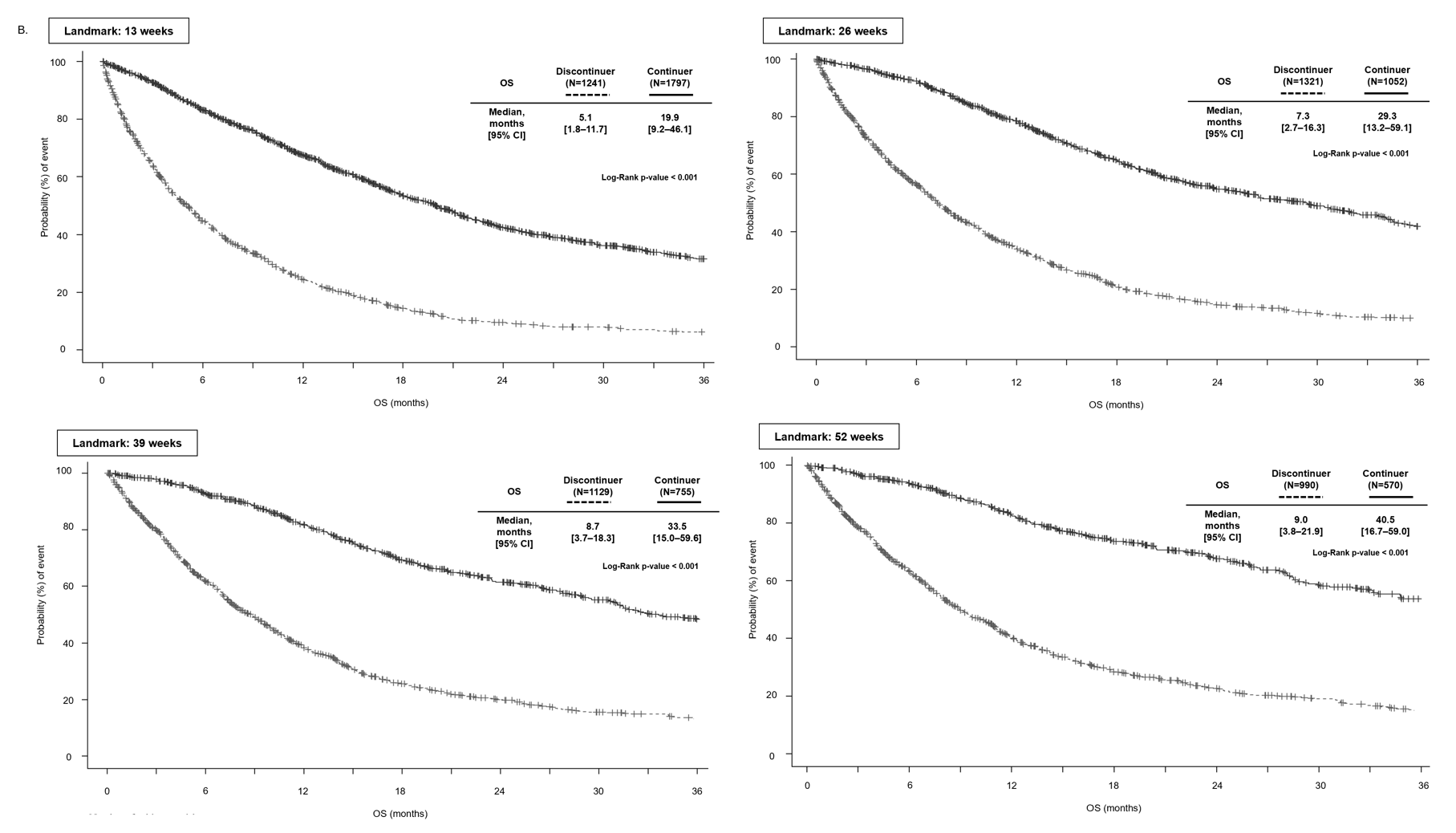


2L+, second line or later; CI, confidence interval; LAM, locally advanced/metastatic; NSCLC, non-small cell lung cancer; OS, overall survival; PFS, progression-free survival.

Supplementary table 1. Retreatment patterns of patients who discontinued index nivolumab

|  |  | **Index nivolumab treatment duration (weeks)** | | |
| --- | --- | --- | --- | --- |
|  | **Overall** | **<13** | **13–25** | **≥26** |
| **Index nivolumab discontinuation, n** | **2,984** | 1,632 | 613 | 739 |
| **Median follow-up from index nivolumab discontinuation, months (Q1–Q3)** | **5.5 (2.3–12.7)** | 3.5 (1.6–8.4) | 7.7 (3.5–15.1) | 10.3 (4.6–19.6) |
| **Retreatment rates following index nivolumab discontinuation, n (%)** | | | | |
| No retreatment or other condition | **1,154 (38.7)** | 687 (42.1) | 171 (27.9) | 296 (40.1) |
| Retreatment or treatment with CT and/or TKI | **1,830 (61.3)** | 945 (57.9) | 442 (72.1) | 443 (59.9) |
| CT and/or TKI | **1,604 (53.8)** | 887 (54.4) | 391 (63.8) | 326 (44.1) |
| ICI rechallenge | **110 (3.7)** | 33 (2.0) | 25 (4.1) | 52 (7.0) |
| ICI resumption | **102 (3.4)** | 25 (1.5) | 23 (3.8) | 54 (7.3) |
| ICI switch | **14 (0.5)** | 0 (0.0) | 3 (0.5) | 11 (1.5) |

2L+, second line or later; CT, chemotherapy; ICI, immune checkpoint inhibitor; Q, quartile; TKI, tyrosine kinase inhibitor.

Supplementary table 2. Patient demographics, clinical characteristics, and treatment characteristics for patients with LAM NSCLC who received nivolumab in 2L (sensitivity analysis)

|  |  | **2L nivolumab index treatment duration (weeks)** | | | | | |
| --- | --- | --- | --- | --- | --- | --- | --- |
|  | **Overall** | **<13** | **13–25** | **26–38** | **39–51** | **52–103** | **≥104** |
| **Population size (N)** | 2,442 | 1,299 | 465 | 199 | 111 | 232 | 136 |
| **Year of 2L nivolumab index initiation, n (%)** | | | | | | | |
| <2017 | 868 (35.6) | 473 (36.4) | 169 (36.3) | 57 (28.6) | 28 (25.2) | 67 (28.9) | 74 (54.4) |
| ≥2017 | 1,574 (64.5) | 826 (63.6) | 296 (63.7) | 142 (71.4) | 83 (74.8) | 165 (71.1) | 62 (45.6) |
| **Age at 2L nivolumab index initiation, years** | | | | | | | |
| Median | 64.1 (57.8–70.6) | 64.1 (57.5–70.5) | 64.5 (58.7–70.5) | 64 (58.7–69.8) | 65.4 (58.8–72.4) | 64.7 (59.5–71.8) | 60.3 (55.2–67.9) |
| **Age ≥75 years at nivolumab initiation, n (%)** | 302 (12.4) | 169 (13.0) | 58 (12.5) | 20 (10.0) | 10 (9.0) | 35 (15.1) | 10 (7.3) |
| **Male sex, n (%)** | 1,720 (70.4) | 921 (70.9%) | 331 (71.2) | 140 (70.4) | 80 (72.1) | 158 (68.1) | 90 (66.2) |
| **Smoking status, n (%)** | 2,368 | 1,258 | 452 | 194 | 107 | 226 | 131 |
| Former smoker | 1,349 (57.0) | 719 (57.2) | 261 (57.7) | 107 (55.2) | 66 (61.7) | 128 (56.6) | 68 (51.9) |
| Smoker | 905 (38.2) | 470 (37.4) | 172 (38.1) | 82 (42.3) | 35 (32.7) | 88 (38.9) | 58 (44.3) |
| Non-smoker | 114 (4.8) | 69 (5.5) | 19 (4.2) | 5 (2.6) | 6 (5.6) | 10 (4.4) | 5 (3.8) |
| **De novo or “relapsed/‌refractory” NSCLC diagnosis type, n (%)** | 2,442 | 1,299 | 465 | 199 | 111 | 232 | 136 |
| *De novo* | 2,002 (82.0) | 1,085 (83.5) | 367 (78.9) | 154 (77.4) | 88 (79.3) | 195 (84.1) | 113 (83.1) |
| Relapsed/ | 440 (18.0) | 214 (16.5) | 98 (21.1) | 45 (22.6) | 23 (20.7) | 37 (15.9) | 23 (16.9) |
| refractory |  |  |  |  |  |  |  |
| **TNM stage at initial diagnosis, n (%)** | 2,406 | 1,280 | 459 | 198 | 109 | 228 | 132 |
| I–III | 732 (30.4) | 365 (28.5) | 159 (34.6) | 74 (37.3) | 32 (29.4) | 66 (28.9) | 36 (27.3) |
| IV | 1,674 (69.6) | 915 (71.5) | 300 (65.4) | 124 (62.6) | 77 (70.6) | 162 (71.1) | 96 (72.7) |
| Not available | 36 | 19 | 6 | 1 | 2 | 4 | 4 |
| **NSCLC histological subtype, n (%)** | 2,442 | 1,299 | 465 | 199 | 111 | 232 | 136 |
| Adenocarcinoma | 1,561 (63.9) | 829 (63.8) | 291 (62.6) | 121 (60.8) | 63 (56.8) | 153 (65.9) | 104 (76.5) |
| **ECOG performance status at 2L nivolumab index date initiation, n (%)** | 1,303 | 704 | 244 | 99 | 54 | 128 | 74 |
| 0 | 258 (19.8) | 102 (14.5) | 57 (23.4) | 30 (30.3) | 18 (33.3) | 37 (28.9) | 14 (18.9) |
| 1 | 737 (56.6) | 379 (53.8) | 150 (61.5) | 51 (51.5) | 27 (50.0) | 78 (60.9) | 52 (70.3) |
| ≥2 | 308 (23.6) | 223 (31.7) | 37 (15.2) | 18 (18.2) | 9 (16.7) | 13 (10.2) | 8 (10.8) |
| **At least one metastatic site at nivolumab initiation, n (%)** | 2,219 (90.9) | 1,202 (92.5) | 416 (89.5) | 174 (87.4) | 100 (90.1) | 201 (86.6) | 126 (92.6) |
| **Location of metastases, n (%)** | | | | | | | |
| Bone | 1,033 (46.6) | 640 (53.2) | 177 (42.5) | 68 (39.1) | 43 (43.0) | 58 (28.9) | 47 (37.3) |
| Brain | 789 (35.6) | 427 (35.5) | 137 (32.9) | 57 (32.8) | 37 (37.0) | 87 (43.3) | 44 (34.9) |
| Contralateral lung | 795 (35.8) | 412 (34.3) | 158 (38.0) | 70 (40.2) | 43 (43.0) | 72 (35.8) | 40 (31.7) |
| Liver | 518 (23.3) | 352 (29.3) | 85 (20.4) | 24 (13.8) | 18 (18.0) | 20 (10.0) | 19 (15.1) |
| **Comorbidities recorded at lung cancer diagnosis** | | | | | | | |
| **Medical history available, n (%)** | 2,330 (95.4) | 1,242 (95.6) | 441 (94.8) | 187 (94.0) | 108 (97.3) | 221 (95.3) | 131 (96.3) |
| **Record of comorbidities, n (%)** | 1,178 (50.6) | 598 (48.1) | 235 (53.3) | 93 (49.7) | 62 (57.4) | 123 (55.7) | 67 (51.1) |
| High blood pressure | 779 (66.1) | 402 (67.2) | 143 (60.9) | 66 (71.0) | 46 (74.2) | 75 (61.0) | 47 (70.1) |
| COPD | 400 (34.0) | 191 (31.9) | 82 (34.9) | 38 (40.9) | 21 (33.9) | 47 (38.2) | 21 (31.3) |
| Diabetes mellitus | 280 (23.8) | 145 (24.2) | 60 (25.5) | 20 (21.5) | 14 (22.6) | 29 (23.6) | 12 (17.9) |
| **PD-L1 screening,* n (%)** | **2,442** | **1,299** | **465** | **199** | **111** | **232** | **136** |
| **PD-L1 screening test result** | | | | | | | |
| Negative, n (%) | 532 (60.0) | 302 (63.6) | 109 (64.5) | 41 (52.6) | 28 (56.0) | 36 (45.0) | 16 (45.7) |
| Positive, n (%) | 331 (37.3) | 161 (33.9) | 57 (33.7) | 30 (38.5) | 21 (42.0) | 43 (53.8) | 19 (54.3) |
| **If positive (≥1%), category of tumor activated cells,** | 310 | 152 | 54 | 26 | 19 | 41 | 18 |
| 1% to 49% expression, n (%) | 191 (61.6) | 95 (62.5) | 36 (66.7) | 13 (50.0) | 15 (78.9) | 26 (63.4) | 6 (33.3) |
| ≥50% expression, n (%) | 119 (38.4) | 57 (37.5) | 18 (33.3) | 13 (50.0) | 4 (21.1) | 15 (36.6) | 12 (66.7) |
| **Lines of therapy received over the entire follow-up, n (%)** | | | | | | | |
| 2 | **1,339 (54.8)** | 744 (57.3) | 200 (43.0) | 85 (42.7) | 55 (49.5) | 150 (64.7) | 105 (77.2) |
| 3 | **605 (24.8)** | 330 (25.4) | 134 (28.8) | 57 (28.6) | 21 (18.9) | 43 (18.5) | 20 (14.7) |
| 4+ | **498 (20.4)** | 225 (17.3) | 131 (28.2) | 57 (28.6) | 35 (31.5) | 39 (16.8) | 11 (8.1) |
| **Line of therapy before index nivolumab, n (%)** | | | | | | | |
| 1 | **2,442 (100)** | 1,299 (100) | 465 (100) | 199 (100) | 111 (100) | 232 (100) | 136 (100) |
| **Median time (Q1–Q3) between first and second lines of therapy, months** | **6.6 (3.9–10.1)** | 5.8 (3.4–9.0) | 7 (4.2–11.4) | 8 (5.3–11.8) | 7 (4.5–10.3) | 8.2 (5.5–12.8) | 7.3 (4.5–12.5) |
| **Median duration (Q1–Q3) of index nivolumab 2L treatment, months** | **2.6 (1.4–6.5)** | 1.4 (0.8–1.9) | 4.1 (3.5–5.0) | 7.1 (6.4–8.1) | 10.1 (9.6–10.8) | 16.4 (14.1–19.8) | 31.3 (26.8–39.4) |
| **Time (Q1–Q3) between LAM NSCLC diagnosis to index nivolumab 2L initiation, median, months** | **8.1 (5.2–12.2)** | 7.4 (4.8–10.7) | 8.6 (5.4–13.6) | 9.4 (6.9–14.3) | 8.4 (5.4–12.9) | 9.8 (6.8–14.8) | 8.7 (5.7–14.8) |
| **1L duration, median (Q1–Q3), months** | **3.3 (2.1–5.3)** | 3.1 (2.0–4.9) | 3.3 (2.1–5.6) | 4.0 (2.2–6.3) | 3.3 (1.9–5.6) | 3.5 (2.2–5.7) | 3.8 (2.1–5.9) |
| **Type of 1L treatment received, n (%)** | | | | | | | |
| Platinum-CT | **2,308 (94.5)** | 1,227 (94.5) | 444 (95.5) | 183 (92.0) | 104 (93.7) | 221 (95.3) | 129 (94.9) |
| Non-platinum-CT | **97 (4.0)** | 51 (3.9) | 18 (3.9) | 12 (6.0) | 5 (4.5) | 7 (3.0) | 4 (2.9) |
| PKI | **16 (0.7)** | 8 (0.6) | 2 (0.4) | 1 (0.5) | 1 (0.9) | 2 (0.9) | 2 (1.5) |
| ICI | **10 (0.4)** | 7 (0.5) | 1 (0.2) | 0 (0.0) | 0 (0.0) | 2 (0.9) | 0 (0.0) |
| Other^†^ | **11 (0.5)** | 6 (0.5) | 0 (0.0) | 3 (1.5) | 1 (0.9) | 0 (0.0) | 1 (0.7) |
| **Nivolumab discontinuers, n (%)** | **1,779 (72.9)** | 956 (73.6) | 373 (80.2) | 154 (77.4) | 79 (71.2) | 145 (62.5) | 72 (52.9) |
| **If discontinued, reason (multiple possible):** | | | | | | | |
| Disease progression, n (%) | **1,191 (66.9)** | 656 (68.5) | 276 (74.0) | 104 (67.5) | 54 (68.4) | 77 (53.1) | 24 (33.3) |
| Doctor’s choice/protocol-driven choice, n (%) | **351 (19.7)** | 170 (17.8) | 61 (16.4) | 32 (20.8) | 13 (16.5) | 42 (29.0) | 33 (45.8) |
| Toxicity, n (%) | **170 (9.6)** | 80 (8.4) | 32 (8.6) | 18 (11.7) | 9 (11.4) | 21 (14.5) | 10 (13.9) |
| Patient’s choice, n (%) | **32 (1.8)** | 21 (2.2) | 3 (0.8) | 0 (0.0) | 2 (2.5) | 4 (2.8) | 2 (2.8) |
| Other, n (%) | **126 (7.1)** | 75 (7.8) | 20 (5.4) | 6 (3.9) | 7 (8.9) | 15 (10.3) | 3 (4.2) |

*Any time during the course of the disease. †Patients in clinical trials and those treated with other monoclonal antibodies. 1L, first line; 2L, second line; COPD, chronic obstructive pulmonary disease; CT, chemotherapy; ECOG, Eastern Cooperative Oncology Group; ICI, immune checkpoint inhibitor; LAM, locally advanced/metastatic; NSCLC, non-small cell lung cancer; PD‑L1, programmed death ligand 1; PKI, protein kinase inhibitor; Q, quartile; TNM, tumor, node, metastasis.

Supplementary table 3. Retreatment patterns of patients who discontinued index nivolumab in 2L only (sensitivity analysis)

|  |  | **2L nivolumab index treatment duration (weeks)** | | |
| --- | --- | --- | --- | --- |
|  | **Overall** | **<13** | **13–25** | **≥26** |
| **2L nivolumab index discontinuation, n** | **1,780** | 957 | 373 | 450 |
| **Treatment rates following 2L nivolumab discontinuation, n (%)** | | | | |
| No retreatment or | **472 (26.5)** | 231 (24.1) | 91 (24.4) | 150 (33.3) |
| other condition | **222 (12.5)** | 177 (18.5) | 20 (5.4) | 25 (5.6) |
| Retreatment or treatment with CT and/or TKI | **1,085 (61.0)** | 548 (57.3) | 262 (70.2) | 275 (61.1) |
| CT and/or TKI | **948 (53.3)** | 518 (54.1) | 235 (63.0) | 195 (43.3) |
| ICI rechallenge | **72 (4.0)** | 19 (2.0) | 16 (4.3) | 37 (8.2) |
| ICI resumption | **58 (3.3)** | 11 (1.1) | 10 (2.7) | 37 (8.2) |
| ICI switch | **7 (0.4)** | NA | 1 (0.3) | 6 (1.3) |

2L, second line; CT, chemotherapy; ICI, immune checkpoint inhibitor; NA, not available; TKI, tyrosine kinase inhibitor.

Supplementary table 4. Survival outcomes of patients treated with index nivolumab in 2L+ and 2L only (sensitivity analysis)

| **Index nivolumab** | **Median OS months [95% CI]** | **OS rate at 1 year, %** | **OS rate at 2 years, %** | **Median PFS months [95% CI]** | **PFS rate at 1 year, %** | **PFS rate at 2 years, %** |
| --- | --- | --- | --- | --- | --- | --- |
| **line of therapy** |  |  |  |  |  |  |
| 2L+ | 10.2 [9.6–10.8] | 42.3 | 25.9 | 2.2 [2.1–2.3] | 16.6 | 9.1 |
| 2L | 10.4 [9.7–11.4] | 47.1 | 26.9 | 2.3 [2.2–2.5] | 17.3 | 9.7 |

2L, second line; 2L+, second line or later; CI, confidence interval; OS, overall survival; PFS, progression free survival.
